# Multi-Color Quantum Dot Tracking Using a High-Speed Hyperspectral Line-Scanning Microscope

**DOI:** 10.1371/journal.pone.0064320

**Published:** 2013-05-22

**Authors:** Patrick J. Cutler, Michael D. Malik, Sheng Liu, Jason M. Byars, Diane S. Lidke, Keith A. Lidke

**Affiliations:** 1 Department of Physics & Astronomy, University of New Mexico, Albuquerque, New Mexico, United States of America; 2 Department of Pathology and Cancer Research and Treatment Center, University of New Mexico, Albuquerque, New Mexico, United States of America; Cornell University, United States of America

## Abstract

Many cellular signaling processes are initiated by dimerization or oligomerization of membrane proteins. However, since the spatial scale of these interactions is below the diffraction limit of the light microscope, the dynamics of these interactions have been difficult to study on living cells. We have developed a novel high-speed hyperspectral microscope (HSM) to perform single particle tracking of up to 8 spectrally distinct species of quantum dots (QDs) at 27 frames per second. The distinct emission spectra of the QDs allows localization with ∼10 nm precision even when the probes are clustered at spatial scales below the diffraction limit. The capabilities of the HSM are demonstrated here by application of multi-color single particle tracking to observe membrane protein behavior, including: 1) dynamic formation and dissociation of Epidermal Growth Factor Receptor dimers; 2) resolving antigen induced aggregation of the high affinity IgE receptor, FcεR1; 3) four color QD tracking while simultaneously visualizing GFP-actin; and 4) high-density tracking for fast diffusion mapping.

## Introduction

Cellular signal transduction is regulated through the interactions and output of membrane receptors. However, the study of molecular-level protein-protein interactions in living cells continues to be a challenging goal in cell biology. While the nanometer-scale distribution of receptors can be observed using electron microscopy (EM), this technique is limited to fixed samples [1]–[6]. Super-resolution microscopy techniques [6]–[9] can also reveal nanometer-scale protein distributions, yet these measurements are inherently slow, making the observation of dynamic events difficult. Therefore, new live cell microscopy techniques are needed in order to determine the role of receptor dynamics in the regulation of cell signaling.

One method for studying the dynamics of membrane components is single particle tracking (SPT) [10], in which a fluorescent or light scattering probe is attached to the component of interest and its position is recorded over time. The probe can be localized with precision much better than the diffraction limit (typically tens of nanometers) by fitting the spatial distribution of light intensity recorded to the point spread function (PSF) of the microscope [11]. Connecting particle coordinates across frames generates particle trajectories that can be analyzed to deduce properties of the probe target or the membrane. Although SPT has been used extensively for characterizing membrane environments [12], it has seen limited use for the study of protein interactions since the localization precision of the probe is greatly reduced when the particle spacing approaches that found in protein dimers or oligomers [13]. Simultaneous tracking of two spectrally distinct probes can be easily achieved using filter-based separation of the emission. Two-color SPT allows the spatially overlapping particles to be separated by emission spectrum, and in the case of isolated, two-color pairs, the localization precision is restored. This concept has been used for the study of interacting membrane proteins in several systems [14]–[18]. Despite its advantages, two-color SPT has several limitations: 1) successful tracking requires each color of probe be present at a density of less than about 1 µm^−2^; 2) same-color dimers are as likely as two-color dimers; and 3) higher order oligomers cannot be easily resolved.

**Figure 1:**
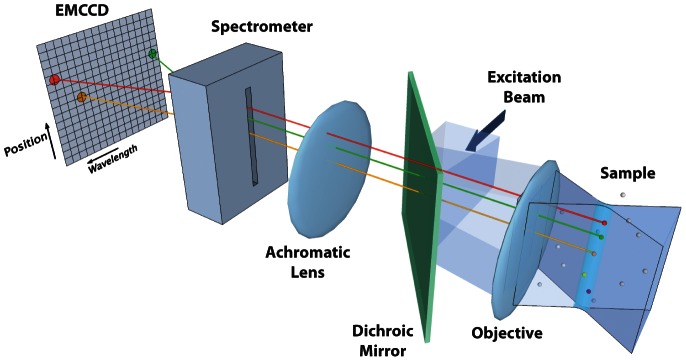
Conceptual diagram of microscope. The excitation beam (optics not shown) is reflected by a dichroic mirror and forms a laser line focused at the sample plane by the objective, concentrating the excitation light to a small volume of diffraction limited width. Here, the white spheres in the sample represent fluorophores that remain mostly in the ground state while the colored spheres denote those which are excited. The emitted light passes through the dichroic mirror and into a spectrometer, which distributes the light onto an EMCCD camera such that each exposure captures information of wavelength and position along the line. The entrance slit on the spectrometer also serves to reject out-of-focus light, providing a semi-confocal ability for imaging at any depth in the sample. A scanning mirror (not shown) advances the line position by one back-projected pixel length on the sample and another exposure is acquired. One hyperspectral “frame” is a reconstructed series of these steps (performed in post processing) to form an image containing *x*, *y*, and *λ*. A time series of these hyperspectral frames is acquired at 27 fps, providing spatial, spectral, and temporal resolution that enables localized single molecule tracking of multiple emitters within a given diffraction limited volume. For a more detailed description, see **Figure 2** **and Text S1**.

Here we describe a high-speed hyperspectral microscope (HSM) that is capable of resolving and tracking eight or more spectrally distinct fluorescent probes. A hyperspectral imaging system collects a region of the spectrum divided into many contiguous channels for each spatial element of the image. Our microscope design is based around the concept of a spectrally-resolved, line-scanning confocal microscope [19], illustrated in **Figure 1**. A laser focused to a line provides high excitation intensity in a region in and around the focal plane of the objective. The line geometry and the use of a confocal slit on the emission path rejects much of the out-of-focus light that is emitted from either fluorophores or sample auto-fluorescence, thereby providing optical sectioning [20]. To create a two-dimensional (2D) hyperspectral image, the line is scanned across the sample plane in discrete steps (along the *x*-axis) and the camera captures a frame at each step. Each single camera capture consists of 64 pixels of 1D spatial information and 128 spectral channels. Therefore, a 2D hyperspectral image consists of a 3D (*x*,*y*,*λ*) data cube collected in these steps. The rate at which complete hyperspectral images can be captured is limited by the readout rate of the electron multiplying charge coupled device (EMCCD) camera and is 27 frames per second (fps) when scans are configured for 32 steps per image (each image is 32×64×128). In this configuration the camera is collecting approximately 900 images per second where each camera image corresponds to a single position of the excitation line. In the application of multi-color SPT (mcSPT), each hyperspectral image is analyzed to find the positions of the fluorescent probes and these positions are used to construct particle trajectories.

We describe the design and performance of our instrument and the analysis process required for mcSPT. We then demonstrate the capabilities of the instrument through several proof-of-principle experiments by applying mcSPT to biological systems of interest in our laboratory. Specifically, we show that: 1) The increased labeling density afforded by mcSPT makes detection of rare and transient receptor interactions more efficient, as demonstrated in observations of Epidermal Growth Factor Receptor (EGFR) dimerization [14]; 2) The multiplexing capability of the HSM can be used for determination of antigen-induced FcεRI aggregate size based on spectral signature and therefore could be used for cluster size correlation with diffusion coefficient [17], [21], [22]; 3) mcSPT can be used while simultaneously visualizing other membrane associated structures, which we demonstrate by performing mcSPT while imaging GFP-actin; and 4) High density tracking facilitated by mcSPT can be exploited for fast diffusion mapping of the cell membrane. Because of the advantages in speed, sensitivity and spectral imaging afforded by our HSM, it is likely that the instrument will find use in a wide variety of biological imaging studies and we briefly discuss a few of these other potential applications.

## Results

### Microscope Design and Performance

**Figure 2:**
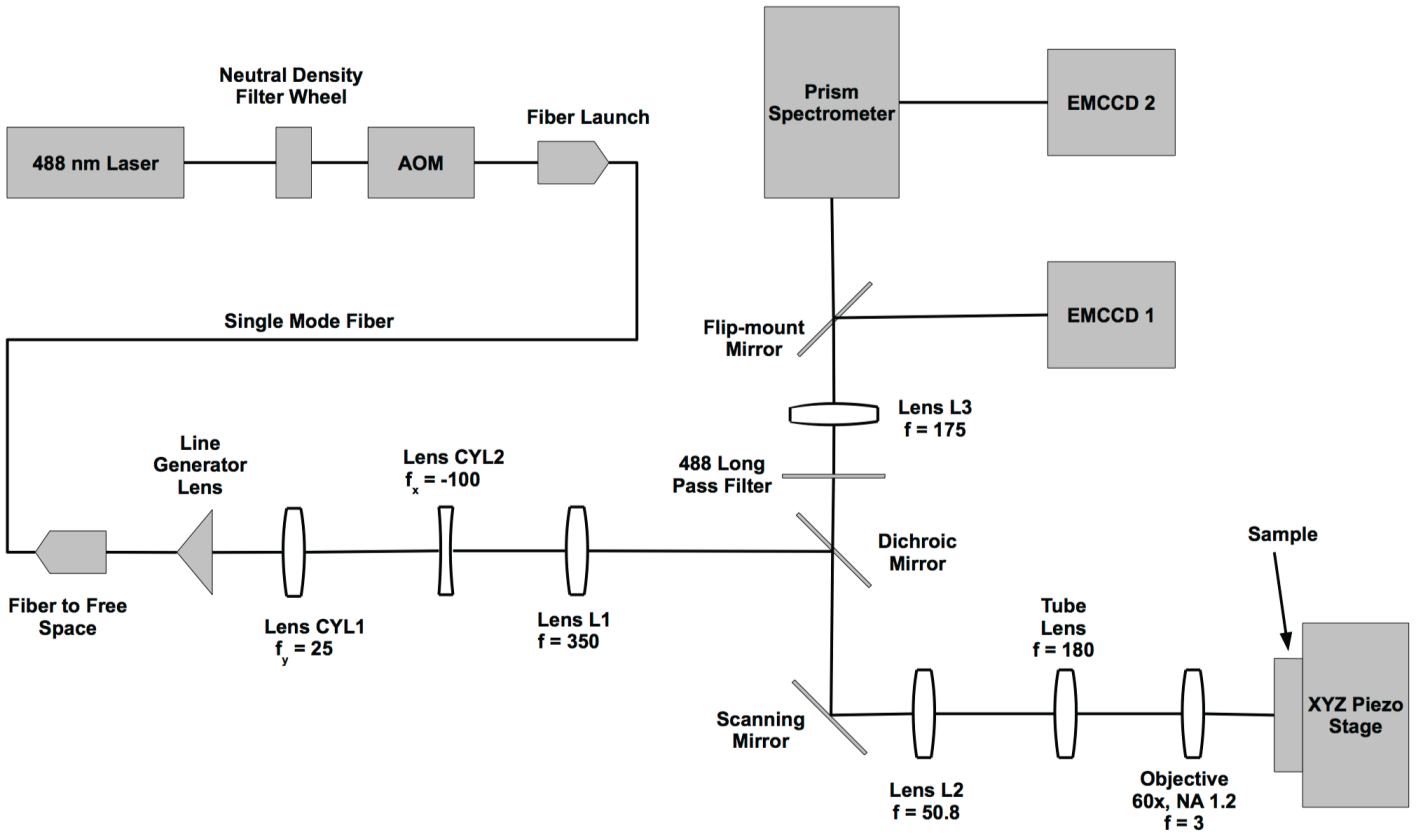
Optical layout. This schematic layout of the laser line scanning hyperspectral microscope displays the key components of the system. Focal lengths are shown in units of mm. A detailed description and parts reference is provided in **Text S1**.

The HSM described here can operate at a frame rate of up to 27 fps for an imaging area of 28 µm^2^ while each spatial pixel collects 128 spectral channels. Our system is designed to span the spectral range from 500 to 750 nm. The system is built around a standard inverted microscope base (Olympus IX71) in order to facilitate live cell imaging. A single 488 nm wavelength laser (which is used to simultaneously excite all spectral species of QDs) is modified with a laser line generating lens (Edmund Optics) and other optics to provide a uniform (vertical) and diffraction limited (horizontal) line at the microscope focal plane. The laser line is scanned across the sample using a galvanometer controlled scanning mirror (Cambridge Research). Emission light is de-scanned and passes through a prism spectrometer and is imaged with a high-speed EMCCD camera (Andor iXon 860). The microscope design layout is provided in **Figure 2** and a more detailed description of the instrument is given in **Text S1**. Synchronization of the camera and scanning mirror is achieved by using the camera as the master clock. Specific digital sequences are pre-programmed in a data acquisition card (NI PCIe-6343) and the camera fire signal is used to trigger the next output state. **Figure S1** shows the overall instrument control schematic, and **Figure S2** provides a few example plots that illustrate the signal timing. When collecting a time series of hyperspectral images (four-dimensional data sets (*x*,*y*,*λ*,*t*)), the camera is set to collect a single kinetic series, and therefore absolute timing is preserved. Data is saved asynchronously as one file for each hyperspectral image.

The localization precision of single fluorophores is proportional to the square root of the number of photons captured [11] and therefore, for throughput efficiency, a prism spectrometer is used as the dispersing element. Prism spectrometers have a higher transmission efficiency over a wide spectral range as compared with diffraction gratings [23], so they are more suitable for this application. The spectrometer is an aplanatic design [24], with a dispersion element that consists of a spherical wedge prism and a spherical concave mirror. The design was optimized using optical design software (Optics Software for Layout and Optimization, Lambda Research Corporation) to minimize distortion. Custom optical components were fabricated (Rainbow Optics, Eugene, Oregon USA) to the specifications of the optimized design. The details of the design (**Figure S3** and **Text S2**) and the spectrometer distortion and dispersion (**Figure S4**) of the design are included in the supporting information. Spectral calibration was performed using a spectral calibration lamp (MIDL® Wavelength Calibration Lamp) and the 543 nm and 633 nm lines from HeNe lasers. There is no observable curvature in the spectral lines with respect to the spatial axis (**Figure S5**).

The measured 4D (*x*,*y*,*z*,*λ*) microscope point spread function (PSF) shows good agreement with that expected for a diffraction limited system (**Figure S6**, **S7**, and **Text S3**). Localization of multi-fluorophore beads (TetraSpeck, Invitrogen) broken into spectral bins of 504–534, 562–611, and 641–712 nm show less than 15 nm of chromatic induced shift anywhere in the image (**Figure S8**).

Custom graphical user interfaces (GUIs) were developed using MATLAB (Natick, Massachusetts USA) for data acquisition and live viewing of hyperspectral images (**Figure S9**). The data acquisition GUI allows for efficient adjustment and optimization of acquisition parameters for experiments. The live viewing GUI provides the experimentalist instantaneous feedback on sample quality and the ability to find a field of view and focus on the sample.

### QD Localization and Trajectory Connection

**Figure 3:**
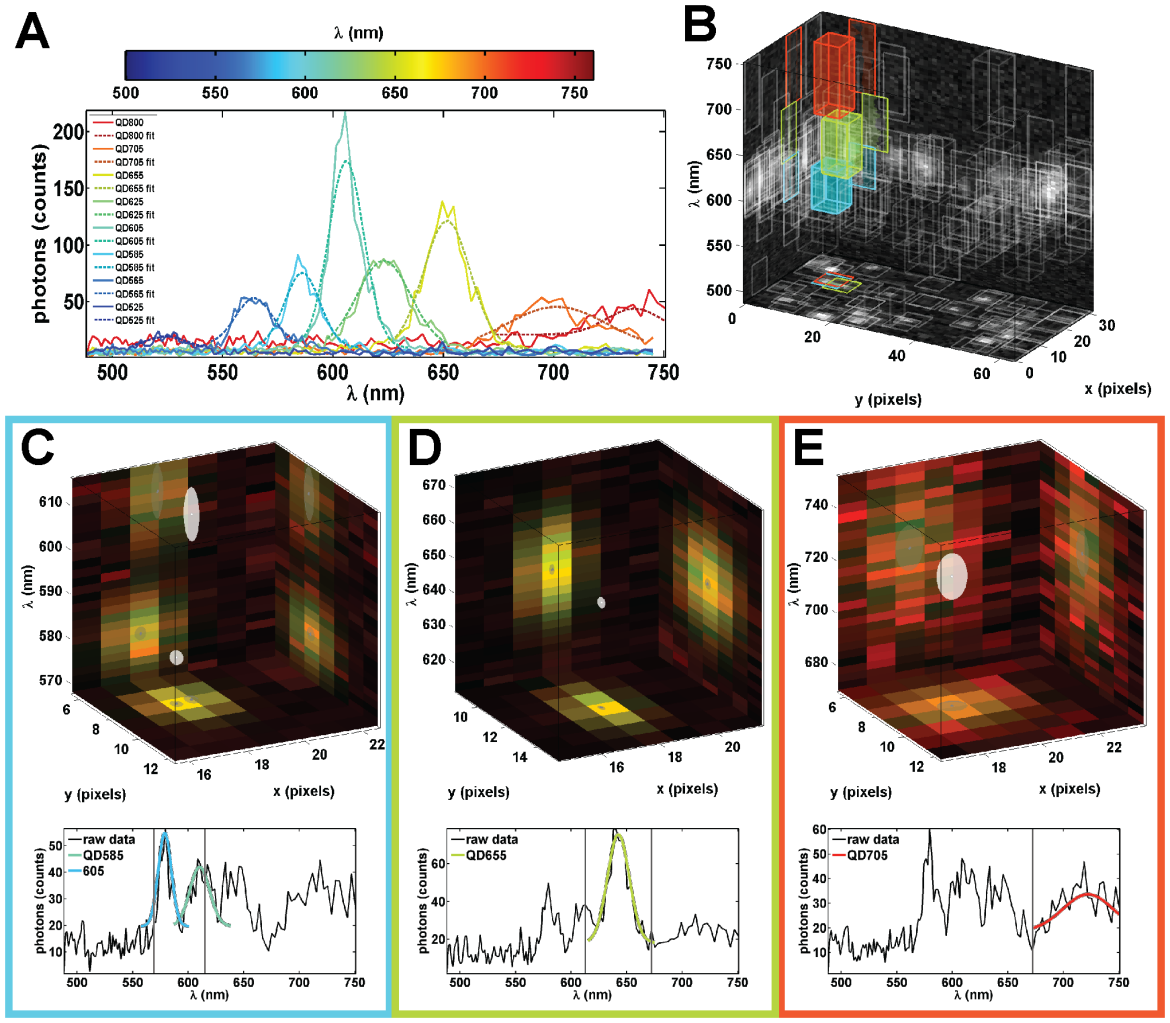
SPT of individual QDs non-specifically adhered to a glass surface. (A) Progressing from blue to red are sum projections of raw data (solid) and Gaussian fits (dashed) for single 525, 565, 585, 605, 625, 655, 705 and 800 nm QDs. (B) 3D representation of a single hyperspectral time frame with 3D boxes representing sub-volumes identified for further particle localization. Sum projections of the data onto each plane are used to represent the raw data (gray scale). Likewise, 2D projections of each 3D box onto each plane are used to highlight the sub-volumes. Fitting results for the colored sub-volumes (color corresponds to the spectral center of the box) in (B) are shown in (C), (D), and (E). In the top figures in (C), (D), and (E) red and green are sum projections of the raw data and fit respectively. Note that the color outlining (C), (D), and (E) correlate with the respective sub-volumes highlighted in (B) with the same color. The localized particles are represented by white ellipsoids in which the radius in each dimension shows 3 standard deviations in the estimated error in the fit using the Cramér Rao Bound and their projection onto each axis is represented by a gray ellipse. The bottom figures in (C), (D), and (E) show raw spectral features (black) and Gaussian fits (color corresponds to fit spectral emission peak). The vertical gray lines represent the spectral cutoffs for independently fit sub-volumes. See **Video S1**.

Single particle tracking is carried out by the localization of single molecules and the subsequent building of trajectories from those localizations [25]. The light emitted from a point source (such as a fluorescently tagged protein) is distributed on the detector according to the PSF of the microscope. The 2D (*x*,*y*) spatial PSF is well modeled by a 2D Gaussian distribution [26] and the emission spectra profiles of QDs are also well modeled by a Gaussian shape (**Figure 3A**). The position of QDs are found by performing a maximum likelihood estimate of spatial position, intensity and spectral peak position using a 3D (*x*,*y*,*λ*) Gaussian PSF model and a Poisson noise model (**Figure S10**, **Text S4**, and **Table S1**). Localizations are performed using an iterative update method [27] that includes the possibility of fitting multiple point emitters within a diffraction limited area [28]. As with previous work [27], [28], the analysis is implemented on graphics processing units in a parallel fashion in order to take advantage of the high computational performance of modern graphical processing units (GPUs) (**Figure S11** and **Text S5**).

To characterize the imaging capability of the HSM, QDs are non-specifically adsorbed onto a glass coverslip and imaged at the fastest scan rate for this geometry. For the field of view collected in these images (64 vertical spatial pixels and 32 line steps; 28 µm^2^), the pixel dwell time was 1.14 ms and the frame rate was 27 fps. The spectral characteristics of individual QDs for 8 colors (commercially available from Invitrogen) are investigated in **Figure S12** and **Table S2**. The procedure for localizing QDs using hyperspectral data is demonstrated with all 8 colors of QDs in a single acquisition (**Figures 3B–E**). See materials and methods for more details. The simultaneous localization of two highly spatially and spectrally overlapping QDs (QD585 and QD605) is highlighted in **Figures 3C and 3F**. Additionally, a total of four highly spatially overlapping QDs are localized simultaneously. This is highlighted by the colored boxes in **Figure 3B** and the corresponding fits in **Figures 3C, 3D and 3E**. The fit precision for each parameter is estimated from the Cramér-Rao Bound (CRB) however, it is important to note that QD blinking during line scanning can contribute to higher than expected error in QD localization in the line-scanning dimension of the data (**Figure S13**). Trajectories are constructed from found QD (*x*,*y*,*λ*) positions (**Video S1**, **Text S6**, and **Figure S14**; manuscript in preparation Relich P, Cutler PJ, Huang F, Lidke KA) [29]. The use of spectral information to build trajectories helps to improve the overall accuracy of the trajectory building process (**Figure S14**).

### mcSPT of Membrane Proteins

#### Observation of Rare, Transient Events

**Figure 4:**
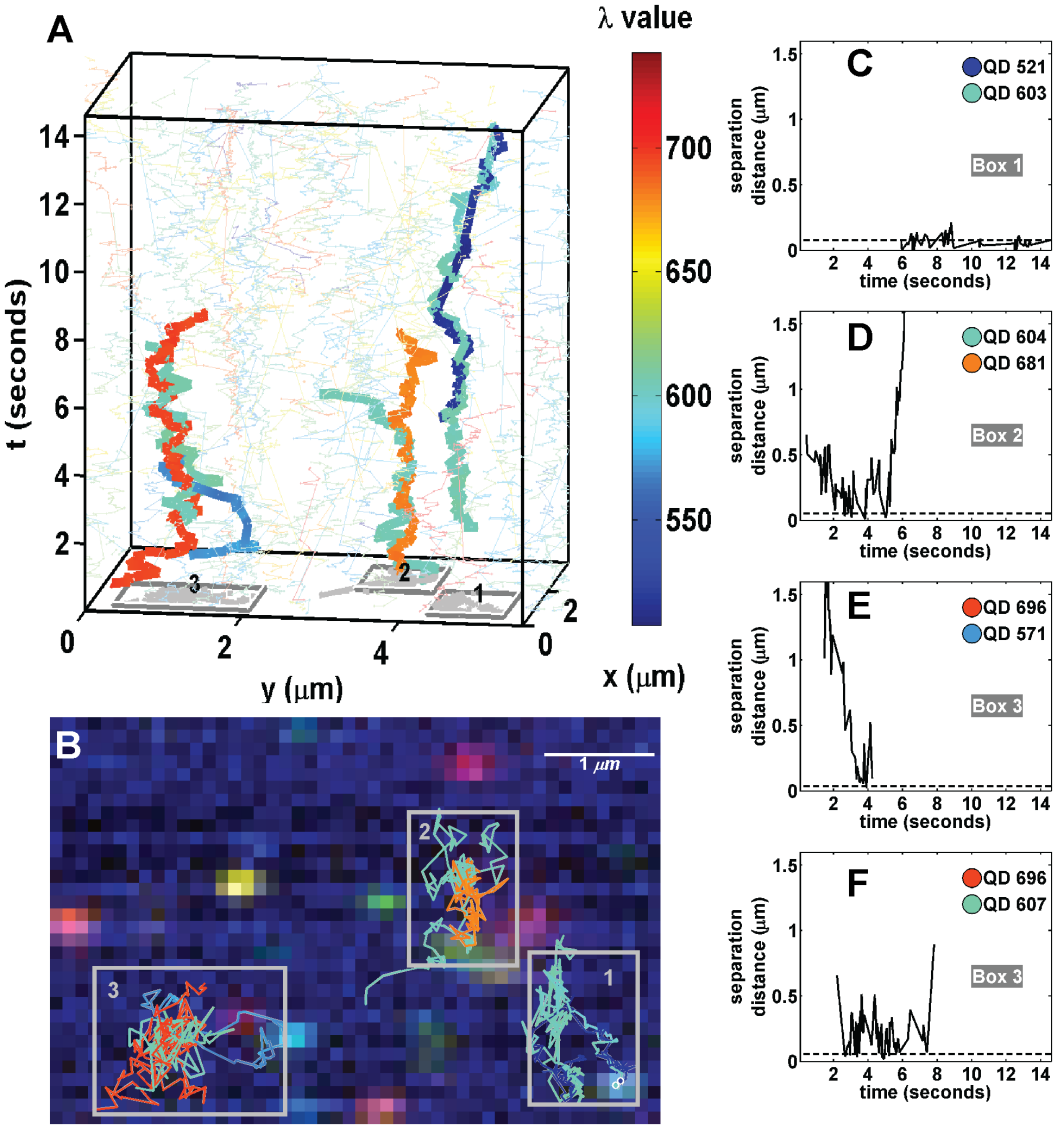
SPT of 8-colors (525, 565, 585, 605, 625, 655, 705, 800 nm) of QD-EGF on live A431 cells. (A) 3D (*x*,*y*,*t*) representation of trajectories. Trajectories are represented by colored lines. Selected interacting trajectories are highlighted by thicker lines. Interactions are grouped by boxes in the xy-plane (identified numerically). (B) Red-green-blue (RGB) representation of raw data for the last time frame with overlaid trajectories and boxes corresponding to (A). Pairwise interaction distances for the selected pairs of QDs are shown in (C), (D), (E), and (F). Note the text identifying how the pairwise interaction distances correspond to the boxes in which the QDs are observed in (A) and (B). See **Video S2**.

Protein-protein interactions can be difficult or impossible to observe with single color SPT since the spacing of proteins at distances less than the diffraction limit make it difficult to discern the difference between merely close proximity and real interactions. Two-color tracking restores the ability to calculate separation and deduce dimerization state. Previously, we used two-color SPT to visualize and quantify EGFR homodimerization under various physiological conditions [14]. These studies required low labeling density such that capturing a sufficient number of dimerization events between two labeled receptors required a large amount of data. Multi-color SPT improves upon two-color SPT by increasing the overall labeling density and thereby increases the potential observation rate of rare events while reducing the probability of same color interactions. Using our HSM, we imaged 8 colors of QD-labeled Epidermal Growth Factor (QD-EGF) bound to EGFR expressed on live A431 cells. **Video S2** shows the analysis results of a data set collected at 27 fps. Highlighted trajectories in **Figure 4A and 4B** show several interacting pairs of QD-EGF-EGFR complexes. Consistent with our previous work [14], we observed long-lived (**Figure 4C**) and transient (**Figure 4D–F**) receptor dimerization events, marked by periods of sustained correlated motion. Note that even in a single time series, multiple interactions are readily identified, demonstrating that the higher labeling density allows for more efficient detection of dimers than with two-color imaging [14].

#### Oligomer Dynamics

**Figure 5:**
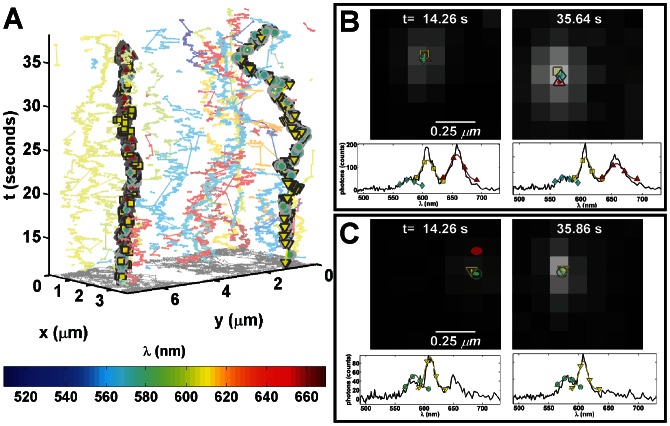
SPT of 5-colors (565, 585, 605, 625, 655) of QD-IgE on live RBL cells. All subfigures are derived from a single acquisition (27 fps) ∼7 minutes after crosslinking with DNP_3_. (A) 3D (*x*, *y*, *t*) depiction of trajectories. The color scheme for the trajectories is dependent on the estimated emission peak of each QD as noted by the color bar. A trimer (565 QD cyan diamond; 605 QD yellow square; 655 QD red up triangle) and dimer (585 QD cyan circle; 605 QD yellow down triangle) are highlighted by their respective symbols. (B) and (C) show localizations for specified time frames. Coloration and symbols correspond to (A). Ellipses in the upper subfigures represent 3 times the localization accuracies over a gray scale projection of the raw data. Red ellipse in the upper left subfigure of (C) is a localized 655 QD that doesn't interact with the dimer. Raw spectra (solid black lines) and Gaussian fit (colored lines with symbols) for individual QDs are shown in the lower subfigures. See **Video S3**.

Previous studies of FcεRI have shown that receptor mobility is a function of antigen dose and valency, and consequently aggregate size [21]. However, in these experiments, the size of aggregates could not be directly determined since a mixture of dark and QD-labeled IgE (QD-IgE) was used to maintain SPT density. Using the HSM to perform mcSPT of QD-IgE-FcεRI on live cells captures not only the dynamics of IgE-FcεRI complexes, but also reveals the number of proteins in the aggregate based on spectral signature. **Figure 5** and **Video S3** show the results of mcSPT of QD-IgE-FcεRI approximately 7 minutes after the addition of a crosslinking agent (DNP_3_). Several long-lived complexes are observed that demonstrate correlated motion. **Figure 5A** highlights the observation of a trimer and dimer. Spectral separation is further beneficial for accurate quantification of diffusion of QD-IgE-FcεRI oligomeric complexes due to their high degree of spatial and spectral overlap (**Figures 5B and 5C**). Note that the localization of the QDs in these complexes would not be possible using single color SPT represented by the gray scale projection in **Figures 5B and 5C**. The red ellipse in **Figure 5C** (upper left subfigure) is a QD655-IgE-FcεRI that comes in close proximity but does not interact with the observed dimer. The spectral features of the QD655-IgE-FcεRI are observed in the raw spectrum (**Figure 5C**; lower left subfigure; solid black line). In a two color SPT experiment, the proximity of a third QD to an observed dimer would disrupt one of the trajectories in the dimer. The diffusion coefficients of the individual trimer and dimer seen in this example are 0.013 and 0.024 µm^2^/s, respectively. These results demonstrate the ability of mcSPT to investigate the dynamics and composition of protein complexes.

#### Interactions with Membrane Components

**Figure 6:**
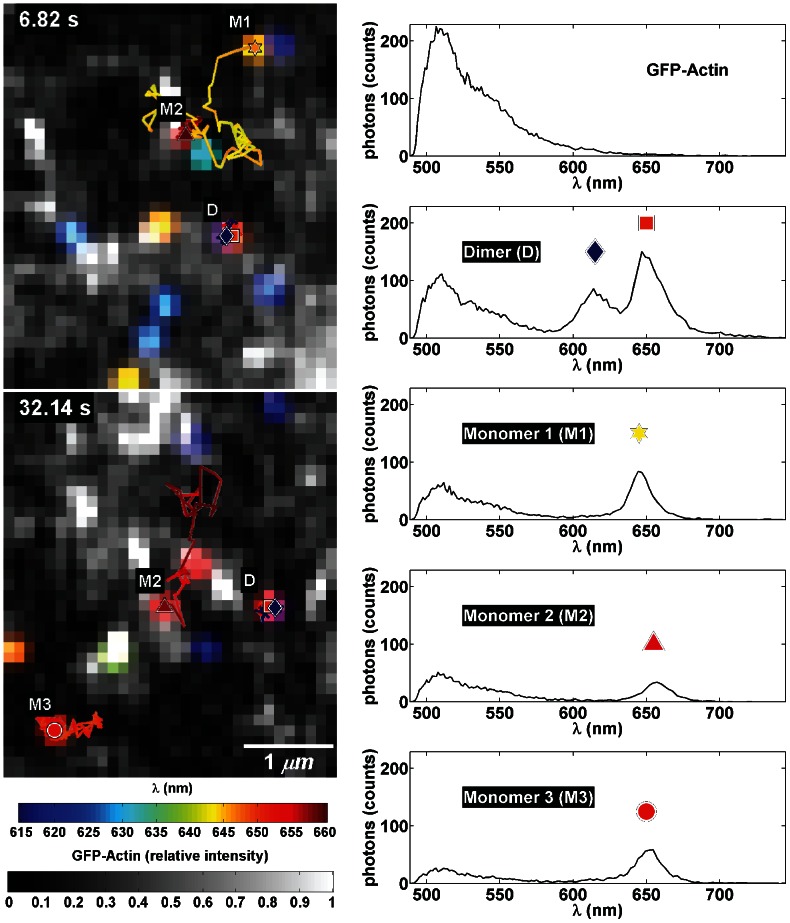
SPT of 4-colors (605, 625, 655, and 705) of QD-IgE-FcεRI on live RBL cells transiently transfected with GFP-actin acquired at 27 fps. (*left*) Two representative frames are shown. The GFP-actin presented in normalized gray scale is the spatially deconvolved portion of the spectrum between 500–570 nm. It is overlaid with Gaussian blobs reconstructed for localized QDs and trajectories for QD-IgE-FcεRI spanning the previous 3.7 s (100 frames; coloration of blobs and trajectories according to emission peak; see color bar). Colored symbols highlight QD-IgE-FcεRI positions at the specified frame, and representative spectra are shown to the right. Text identifiers (D, M1, M2, and M3) are used to indicate monomers (M#) and dimer (D) trajectories. See **Video S4**.

The dynamics of membrane proteins may be influenced by membrane-associated structures such as actin [17], clathrin [30] or other microdomains [31] and interaction with such structures could be correlated with oligomerization state. Our HSM allows the simultaneous observations of several colors of QDs at the same time as other fluorescent probes that can be excited efficiently with 488 nm light, such as GFP. Here we demonstrate mcSPT using 4 colors of QD-IgE-FcεRI (605, 625, 655, and 705 nm) while simultaneously imaging GFP-actin. Imaging was performed on rat basophilic leukemia (RBL-2H3) cells transiently transfected with GFP-actin. **Figure 6** (**Video S4**) provides observations that highlight several advantages of this technique. Comparison of the actin structure in the top and bottom images demonstrates how dynamic the actin structure is over the time course of this acquisition. Actin corralling of QD-IgE-FcεRI is also observed (red triangle and red circle in the bottom image). These results are consistent with previous reports of actin corralling first observed by tracking QD655-IgE-FcεRI with respect to GFP-actin [17]. However, these previous studies were limited to tracking in total internal reflection fluorescence (TIRF) at the adherent surface since wide field imaging of actin structures at the apical surface was insufficient and point scanning confocal was too slow for tracking. Using the HSM, we can perform fast confocal imaging while simultaneously tracking single quantum dots. Moreover, the increased number of spectrally distinct QDs allow for tracking of many receptors simultaneously such that their interactions within the actin domains can be observed. The mobility of the dimer complex (0.004 µm^2^/s; purple blue diamond and red square, top and bottom) is less than that of highlighted monomers (0.044 µm^2^/s). A transition of the mobility of a monomer from relatively immobile (red triangle, top) to relatively mobile (red triangle, bottom) is observed. Crossing trajectories are appropriately connected due to the spectral information (red triangle and yellow star).

### Fast Membrane Diffusion Mapping

**Figure 7:**
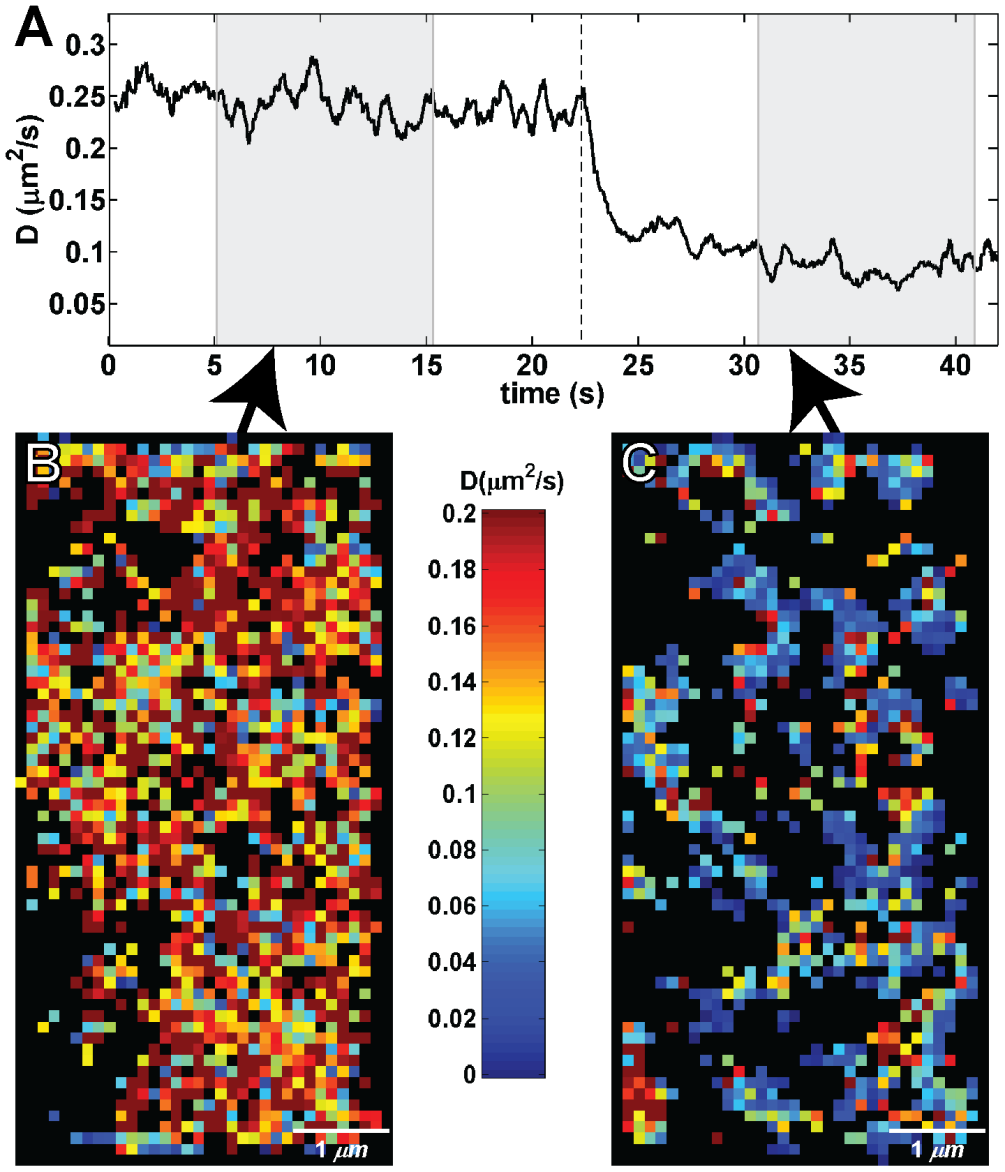
High density single particle tracking (QD 525, 565, 585, 605, 625, 655, 705, and 800). (A) Shows the instantaneous diffusion for a mcSPT experiment with an acquisition rate of 27 fps of QD-IgE. 1 µg/ml of DNP-BSA was added ∼20 seconds into the acquisition (vertical dashed line). (B) and (D) show spatial maps of diffusion coefficients (estimated diffusion coefficient for each spatial bin is defined by the color scheme indicated in the color bar; red faster, blue slower, black insufficient sampling) for the same experiment shown in (A) estimated using all squared displacements observed between ∼5–15 seconds (B) and ∼30–40 seconds (C). A maximum likelihood estimator is used to estimate the diffusion coefficient (manuscript in preparation Cutler P J, Relich P, Lidke KA). See **Video S5**.

Labeling with multiple colors enables SPT at densities of several particles per square micron. By binning squared displacement both temporally and spatially, high-density mcSPT can be used to probe local receptor mobility [32]. The uncertainty in the diffusion coefficient scales as approximately D*(A*ρ*n)^−1/2^ where *D* is the diffusion coefficient, *A* is area, *ρ* is particle density and *n* is the number of time frames (**Text S7**); therefore, higher density allows better spatial and/or temporal resolution in the diffusion map. In a similar manner, high density mcSPT also allows global changes in protein diffusion to be observed quickly. To demonstrate this, a high density mcSPT experiment was devised in which the relative labeling density for 8 colors of QD-IgE-FcεRI was refined to provide similar high density labeling for each color of QD. The labeling density achieved here is ∼2.4 particles per µm^2^ which to the authors' knowledge is higher density than any previously reported SPT method. Crosslinker (DNP-BSA) was added ∼20 seconds into the acquisition and a corresponding change in the estimated instantaneous diffusion coefficient is observed (**Figure 7A**). Due to the increased labeling density, this instantaneous change in mobility observed from a single acquisition of a single cell correlates well with that observed in a previous study that required the combined analysis of multiple (∼10) SPT experiments of single color of QD-IgE [17], [21]. Similarly, squared displacements can be binned both temporally and spatially to build diffusion maps in relation to QD-IgE-FcεRI. In **Figures 7B and 7C**, squared displacements are binned in 280 frame temporal bins (∼10 seconds) and 1 pixel by 1 pixel spatial bins (0.116 µm by 0.116 µm). Changes in diffusion maps subsequent to the addition of crosslinker are apparent upon comparison of **Figures 7B and 7C** (**Video S5**).

### Other Imaging Modalities

Our fast, line scanning HSM has the potential to be used for many other imaging modalities that are not explicitly demonstrated. Some of these modalities are variants of combinations of the demonstrated approaches. mcSPT could be performed to image several types of proteins labeled with various permutations of QD colors. (e.g. two colors on one membrane receptor and two on another). mcSPT could also be performed simultaneously with more than one additional marker, such as both GFP and yellow fluorescent protein (YFP), which could be separated from each other using multivariate curve resolution (MCR) [22].

Since the instrument is a line scanning system with optical sectioning capability, the microscope can be used to obtain hyperspectral *z*-slices of a sample. Using the fast scanning ability and a piezoelectric stage, our HSM can make three dimensional observations of samples with hyperspectral information, providing 5D data sets with spatial (*x*,*y*,*z*), spectral (*λ*), and temporal (*t*) dimensions. A demonstration of live cell, 5D imaging of three cellular markers is shown in the (**Figure S15** and **Video S6**).

Given the ability of the instrument to image single molecules with high temporal and spectral resolution, the HSM could be used with a variety of probes that report on local environment via spectra [33]–[36], for single-molecule, multi-color Foerster resonance energy transfer (FRET) studies [37]–[39] or for studying the fluorophores themselves, particularly QDs [40]–[42].

Finally, the instrument could also be used for more typical hyperspectral imaging tasks, such as imaging samples labeled with many spectrally overlapping fluorophores [43], and imaging spectral shifts of native fluorescence in organisms [22].

## Discussion

Here we have detailed the development of a novel hyperspectral microscope that provides an unprecedented combination of speed, sensitivity and a spectral detection. Our HSM design provides acquisition rates of 27 fps over a 28 µm^2^ field of view with 128 spectral channels. These features allow the determination of stoichiometry and dynamics of small oligomers, which cannot be measured using any other currently available techniques. We have demonstrated these capabilities of our HSM by multi-color single QD tracking and diffusion mapping of membrane protein dynamics. While EM or super-resolution techniques do provide a nanometer-scale image of protein distributions and clustering, they cannot provide information on protein diffusion or clustering dynamics. One variant of super-resolution that captures dynamics uses SPT of sequentially photo-activated fluorescent proteins [32], but it still requires long acquisition times to build up a diffusion map and cannot track a single protein or aggregate for more than a few seconds and cannot be used to identify interactions.

The maximum speed is limited by the EMCCD readout rate and is achieved by imaging only 64 of 128 pixels in the vertical shift direction. In this configuration, the maximum frame rate scales directly with the image area. A higher frame rate or larger field of view could be achieved by rotating the camera such that spectral binning in the vertical shift direction would increase frame rate. This would most benefit fast, 3D imaging of conventional fluorophores. We estimate that the imaging area could be increased by a factor of two while maintaining similar signal to noise levels with a state-of-the-art scientific complementary metal oxide (sCMOS) camera [44], [45]. However, pixel-to-pixel noise and gain variation of sCMOS cameras may complicate particle localization and MCR analysis [46].

Several other spectral imaging methods could be envisioned for performing mcSPT. In principle, a widefield microscope could be used with a series of dichroic and emission filters to setup an arbitrarily large number of spectral channels. This approach has been used for two color [14] and four color imaging [47]. However, for image registration better than 20 nm, which is required for studying protein-protein interactions, careful mapping between all channels must be periodically performed using multispectral reference beads. In contrast, our spectrometer system provides less than 15 nm shift in spatial localization due to chromatic aberration from 500 nm to 750 nm (**Figure S8**). Another recently described approach for wide field, fluorescence spectral imaging uses an image mapping spectrometer [48]. This apparatus would seem not to suffer from channel registration problems and although has only been demonstrated at ∼7 fps, would in principle be capable of video rate imaging. Of course, these wide field approaches do not provide a mechanism for rejecting out of focus light and could therefore suffer as compared to a line-scanning system when imaging in an environment with high cellular auto-fluorescence or other background.

Spectral imaging capabilities can be found on many commercial point scanning microscopes as well as custom built point-scanning hyperspectral systems [23], [43], [49]. However the finite lifetime of fluorophores limits photon yield even at arbitrarily high laser power and leads to a practical limit for SPT in point scanning systems. For a region of interest that is 128×128 spatial pixels, with a desired localization precision of ∼10 nm, the maximum point scanning frame rate is ∼2 fps and the maximum line scanning frame rate is ∼250 fps (**Text S8**). In terms of achieving a maximum area scanned for a given specific frame rate of 30 fps (33 ms per frame) and with the same 10 nm localization precision, these limits are ∼512 um^2^/33 ms for organic fluorophores (τ = 2 ns) and as low as 51 um^2^/33 ms for QDs (τ = 20 ns) for a point scanning system. The latter value is fairly close to the area covered by this instrument when acquiring at 30 fps, and does not include consideration of spectral data acquisition. In addition, any non-linear photobleaching, photo-destruction or long-lived dark states would further reduce these values in practice. In comparison, a line-scanning system has the advantage of parallel observations along the spatial dimension and therefore gives an additional factor equal to the number of pixels that are simultaneously observed.

QDs have a broad excitation spectrum, narrow emission spectrum and high photo-stability. All of these properties are taken advantage of for mcSPT using our HSM. However, QDs do have practical limiting properties [40] such as fluorescence intermittency, the potential for non-fluorescent QDs [50] and a difficultly in producing monovalent probes [51]. For example, the presence of non-fluorescent QDs leads to the possibility of underestimating the oligomer size of complexes in mcSPT experiments. Consideration must also be taken when using biotin-protein∶streptavidin-QD conjugation schemes since unlabeled protein could also lead to underestimation of oligomer size. Consequently, the oligomers identified in **Figures 5** and **6** should be considered as minimal dimers and trimers. Contribution from non-biotynilated protein can be minimized by purification on streptavidin resin prior to conjugation with the QDs. The dark fraction of QDs for each spectral species can be measured and used in a statistical analysis of the properties under study as a function of oligomer size. mcSPT could also be performed with other probes that may have overlapping spectra, such as non-blinking QDs [52], [53], ‘Cornell dots’ [54] or other systems that use combinations of organic fluorophores and could have the benefit of reduced or non-existent fluorescence intermittency.

In summary, we have developed a high-speed hyperspectral microscope and the required analysis software to perform simultaneous SPT of up to 8 spectrally distinct QDs at video rate. We have provided a description of the instrument, software, and several proof-of-principle applications of the HSM. The applications presented in this manuscript (mcSPT, mcSPT with GFP background, high density mcSPT, and 3D scanning) highlight how the HSM can provide unique insight into dynamic cellular events.

## Materials and Methods

### Reagents

Purification [55] of mouse monoclonal anti-DNP IgE and preparation [17] of functionally monovalent QD-IgE was previously reported. Streptavidin conjugated quantum dots were purchased from Invitrogen either individually (525-Q10141MP, 565-Q10131MP, 585-Q10111MP, 605-Q10101MP, 625-A10196, 655-Q10121MP, 705-Q10161MP, 800- Q10171MP) or in the Qdot® Streptavidin Sampler Kit (Q10151MP). Biotin-X, SSE used for monovalent biotinlylation of IgE for linkage to streptavidin QDs was purchased from Invitrogen (B6352). Biotin was purchased from Invitrogen (B1595). CellMask™ orange plasma membrane stain was purchased from Invitrogen (C10045). Multivalent DNP-BSA was purchased from Invitrogen (A23018). DNP_3_ (manuscript in preparation Mahajan A, Barua D, Cutler P, Lidke DS, et al.), which is a small trimer of peptides that presents three DNP moieties for crosslinking DNP-specific IgE, was synthesized by AnaSpec (Fremont, CA). Plasmid used for transfection of GFP-actin into RBL-2H3 cells was purchased from Clontech (pAcGFP1-Actin 632453). Biotinylated EGF at a 1∶1 ratio was purchased from Invitrogen (E-3477). The EGFR inhibitor PD153035 was purchased from Calbiochem (234491).

### Imaging of QDs Non-specifically Adhered to Glass

The quantum dots (Invitrogen Corporation Qdot® 525, 565, 585, 605, 625, 655, 705, and 800) were each diluted to 50 pM in 1× phosphate buffered saline (PBS) and then mixed together in equal volumes. 300 ul of this mixture was then added to each well in an 8-well chamber. 10 ul of 2 M NaCl (aqueous) was then added to each well and then incubated at 4°C for up to 8 hours to allow the quantum dots to attach to the glass. The solution was then replaced by 1× PBS. 250 frames of data (providing *x*, *y*, and *λ* information) were acquired at 27 frames/sec over an area of 28 µm^2^ (the intended scan rate for our typical live cell imaging) with an excitation laser line intensity of ∼1000 W/cm^2^.

### Image Acquisition for SPT Experiments on Live Cells

Samples were maintained at 34–36°C using an objective heater (Bioptechs, Butler, PA, USA) for imaging. For an acquisition rate of 27 fps, a region of interest (ROI) of 3.7 µm in *x* and 7.4 µm in *y* is acquired. This ROI consists of 32 steps (*x* dimension; ∼0.116 µm steps) of the line across the sample by the scanning mirror and 64 pixels (*y* dimension; ∼0.116 µm pixel size) along the line.

### Single Particle Tracking (Preprocessing, Localizations, and Trajectory Building)

Immediately prior to the acquisition of each HSM image, 1000 frames of dark images were acquired for background correction. The mean dark image was subtracted from each frame of the HSM image. The spectrometer was aligned so that several columns of spectral pixels are dark due to a long pass filter.. The mean value of a dark column of pixels was subtracted from the SPT image to correct a baseline offset. The HSM image was then multiplied by a calibrated gain factor [56] to convert arbitrary pixel counts to photons.

The steps in SPT in an HSM image are similar to steps used in traditional SPT algorithms: image segmentation, single particle localization, and trajectory building. Uniform and maximum filtering procedures outlined in previous work by Huang et. al. [28] for two dimensions are expanded to three dimensions (*x*,*y*,*λ*) and used to identify centers for sub-volumes in which to localize single molecules. The empirically determined sub-volume size used for single molecule localization in this work was 25 spectral pixels by 7 spatial pixels by 7 spatial pixels. A 3D Gaussian PSF/spectral model and Poisson noise model (**Figure S10** and **Text S4**) were used to model the spatial and spectral features of individual QDs. The variance in the parameters estimated by the localization algorithm converges to the CRB (**Figure S10B**). Multiple 3D Gaussians were used to fit multiple spatially and spectrally overlapping QDs (**Figure S11** and **Text S5**). An artifact of using a line-scanning instrument is that blinking during the scanning of a single QD causes errors in QD localization in the scan direction (**Figure S13**). Typically, each sub-volume was fit using 1–4 emitter models. Accurate fits were then selected using an empirically derived filtering scheme (**Text S4**). Trajectories are built from filtered localizations using a cost matrix approach to form a linear assignment problem [29] (**Text S6**).

### Quantification of Trajectory Mobility

Diffusion coefficients (*D*) for groups of trajectories (**Figures 5** and **6**) were determined by fitting a line to the first 3 points of the ensemble mean squared displacement. A maximum likelihood estimator based on independent jump analysis (manuscript in preparation Cutler PJ, Relich P, Lidke KA) is used to estimate the instantaneous *D* and diffusion mapping (**Figure 7**). For the instantaneous *D*, individual jumps were grouped temporally using a sliding window of 14 frames (∼0.5 s) according the temporal midpoint of the observations. For diffusion mapping, individual jumps were binned spatially according to camera pixel sampling (∼116 nm in each dimension) and temporally according to the midpoint of the temporal jump using a sliding window of 280 frames (∼10 s).

### Cell Culture

A431 human epithelial carcinoma cells were cultured and passaged in Dulbeccos' modified eagle medium (DMEM, Sigma-Aldrich), with 10% fetal bovine serum (FBS, Invitrogen), penicillin, and streptomycin [14]. RBL-2H3 cells were cultured and passaged using MEM (Gibco® 11095-080) supplemented with penicillin-streptomycin, L-glutamine, and 10% FBS [21], [30]. For all experiments using GFP-actin, RBL-2H3 cells were transiently transfected by electroporation with an Amaxa Nucleofactor II using program T-020. Transiently transfected cells were used within 24–48 h of transfection. For all image acquisitions, cells were plated in Lab-Tek 8-well chambers (Thermo Scientific, 177402) approximately 24–48 hours prior to the experiment at ∼2×10^4^ cells per well.

### Cell Treatment for SPT of QD-EGF

Procedures for labeling A431 cells with QD-EGF were adapted from previous work [14]. Cells were serum starved in the presence of 1 µM PD153035 for several hours prior to imaging. PD153035 (1 µM) was included in imaging buffer throughout the remainder of the experiment to prevent receptor endocytosis. Tyrodes solution with 0.1% BSA and 20 mM glucose was used as an imaging buffer. Media was exchanged with 200 µl of imaging buffer prior to imaging. Immediately prior to data acquisition, 100 µl of 12 pM (concentration for each individual color) of QD-EGF (final concentration of 4 pM) equilibrated at 37°C was added to the well to be imaged and data was acquired within 10 minutes.

### Cell Treatment for SPT of QD-IgE

Procedures for labeling RBL-2H3 cells with QD-IgE for SPT were adapted from previous work [17]. For mcSPT experiments, cells were incubated in 200 µl of Hanks' balanced salt solution (HBSS) with 1 nM of each color of QD-IgE simultaneously for 15 min at 37°C. For the experiment, cells were incubated in 200 µl of HBSS with optimized concentrations of each color of QD-IgE (2 nM of 605, 625, and 655; 4 nM of 565 and 585; 10 nM of 525, 705, and 800) for 15 min at 37°C. Final volume of HBSS in each well was 200 µl. All SPT experiments were performed within 1 hour of labeling. For the mcSPT experiment (**Figure 5**), 100 µl of crosslinker (DNP_3_; final concentration of 1 nM) equilibrated at 37°C was added to the well ∼7 minutes prior to the collection of the acquisition. For the high density mcSPT experiment (**Figure 7**), 100 µl of crosslinker (DNP-BSA; final concentration of 1 µg/ml) equilibrated at 37°C was added ∼20 seconds into the image acquisition.

### 3D-scanning of Live Cells

RBL-2H3 cells were incubated in 200 µl of 5 nM QD-IgE (655) in HBSS for 30 minutes at 37°C followed by 2 washes with HBSS. The cells were incubated with 200 µl of 5 µg/ml CellMask orange for 3 min at 37°C followed by 3 washes with HBSS. Imaging was performed immediately after labeling. Crosslinker (DNP-BSA; final concentration 10 µg/ml) was added ∼60 seconds into the image acquisition. In order to assure rapid mixing of crosslinker and to prevent focal drift, 100 µl of crosslinker stock at 3× final concentration in HBSS preheated to 37°C was added (contains 200 µl HBSS).

*Z*-stacks covering an ROI of 14.8 µm in *x*, 14.8 µm in *y*, and 7.0 µm in *z* were acquired at a rate of 7.72 seconds per *z*-stack. Each z-slice for this ROI consists of 128 steps (*x* dimension; ∼0.116 µm steps) of the line across the sample by the scanning mirror and 128 pixels (*y* dimension; ∼0.116 µm pixel size) along the line to make each *z*-slice, and each *z*-stack for this ROI contains 29 *z*-slices with a step size of 0.25 µm. In order to conserve CPU memory for storage and post-processing, the data was binned into 32 spectral bins instead of 128. It is important to note that altering the number of spectral bins does not significantly alter the acquisition time with the current camera configuration. A total of 50 *z*-stacks were acquired giving a total acquisition time of ∼6.5 min. *Z*-stacks were processed using 3D deconvolution. The experimentally measured PSF was used with the built-in Matlab deconvolution algorithm *deconvlucy*. An isosurface rendering was then used to represent the data.

## Supporting Information

Video S1 **SPT on glass.** SPT of 8-colors of QDs non-specifically adsorbed on glass. (top) RGB representation of the raw data with overlaid trajectories (coloration of trajectories are according to color bar in **Figure 3**). The white box is approximately representative of the spatial region for the highlighted sub-volumes in **Figure 3B–E**. (bottom) Spectrum for the ROI in the white box. This movie accompanies **Figure 3B–E**.
(MP4)

Video S2 **Transient interactions.** Transient (boxes 2 and 3) and stable (box 1) interactions are observed for SPT of 8-colors of QD-EGF on a live A431 cell. This movie is a temporal animation of **Figure 4**.
(MP4)

Video S3 **Oligomer dynamics.** Oligomers are observed after crosslinking QD-IgE-FcεRI with DNP_3_ on a live RBL-2H3 cell. This movie highlights a dimer (top; circle and down triangle) and a trimer (bottom; diamond, square and up triangle). Representative spectra for the highlighted ROIs are shown to the right. This movie accompanies **Figure 5**.
(MP4)

Video S4 **Observation of GFP-actin and QD-IgE-FcεRI.** Simultaneous observation of GFP-actin (gray scale) and SPT of 4-colors of QD-IgE-FcεRI on a live RBL-2H3 cell. This movie accompanies **Figure 6**.
(MP4)

Video S5 **Membrane diffusion mapping.** High density mcSPT of QD-IgE-FcεRI on a live RBL-2H3 cell. (upper left) RGB representation of localized particles and trajectories (color bar below indicates emission peak for localizations and trajectories). Trajectory positions observed in the previous 10 frames (∼0.36 s) are shown. (upper right) Diffusion map relative to QD-IgE-FcεRI (color bar below indicates estimated diffusion coefficients). (bottom) Instantaneous diffusion coefficient corresponding to **Figure 7a** with a moving window indicating the temporal window used to determine the diffusion map. Upon the addition of DNP-BSA, the window changes from red to green (text indicator in the upper right of the movie also changes color) marking the addition of DNP-BSA. This movie accompanies **Figure 7**.
(MP4)

Video S6 **3D scanning of live cells.** Isosurface representation for 3D scanning of a live RBL-2H3 cell. DNP-BSA was added ∼60 s into the acquisition as indicated by the text at the top of the movie. Cells are labeled with Cell Mask orange (blue), Actin-GFP (green), and QD-IgE-FcεRI (red). This movie accompanies **Figure S15**.
(MP4)

Figure S1 **Instrument control schematic.** The fire out signal from the camera is used as the clock to advance the scanning mirror, stepping through a list of digital words provided to the scanning mirror control board. The acousto-optic modulator (AOM) controller accepts analog DC voltage input and controls the RF power output to the AOM's piezoelectric transducer. The flip-mount controller toggles the mirror position with a single 5V pulse. The *XYZ* Piezo-stage controller applies a *z*-position change based on analog DC voltage input and returns position information to an analog voltage input on the data acquisition board. The secondary camera (used for widefield viewing to center a sample or select ROI) uses a single USB connection.
(PNG)

Figure S2 **Signal timing.** (A) As the camera finishes an exposure the falling edge trigger is used as a clock to advance the mirror scan angle by one step. A frame transfer mode EMCCD camera is used, so readout occurs during exposure. The mirror step response time actually exceeds the frame transfer interval, but is much less than a single exposure, such that mirror motion is negligible during acquisition. (B) Scans are completed after a set number of steps, where the mirror angle is returned to the starting position. (C) In fast 3D scans, as a single scan is completed the z stage is translated by one step. (D) As the *z* steps are completed through the depth of the region of interest, the *z* stage is returned to the starting position to begin another pass.
(PNG)

Figure S3 **Spectrometer design.** A more detailed discussion of the principles of the spectrometer design is in **Text S2**. (A) Relative positions of each spherical surface of the prism spectrometer. S1 (red solid circle) and S2 (blue solid circle) are refractive surfaces of the spherical prism, S3 (cyan solid circle) is the reflective surface of the concave mirror. The dotted circles are conjugate aplanatic surfaces of S1 and S2. (B) Relationship of conjugate aplanatic surfaces (dotted circles) of the refractive spherical surfaces (solid circles). (C) 3D drawing of the spectrometer. (D) Spectrometer design in OSLO (Optics Software for Layout and Optimization).
(PNG)

Figure S4 **Spectral distortion and dispersion.** The distortion and dispersion curves are generated from OSLO. (A) Distortion shows offset of the true image position from the ideal image position at various off axis points. In the HSM setup, the maximum distance from the axis is 1.5 mm, so the designed maximum distortion is 0.4 µm, which is less than 2% of CCD pixel size (24 µm). (B) Lateral shift shows the dispersion, designed to span a total of 3 mm from 500 nm to 800 nm. It shows dispersions in four relative heights (distance from the optical axis), the four curves are right on top of each other, so the spectral lines are straight, which also indicates a minimal distortion.
(PNG)

Figure S5 **Spectral calibration.** (A) Image of the calibration lamp on the camera (the vertical axis is the spectral dimension). The calibration lamp is a LightForm multi-ion discharge lamp (MIDL), the slit width is 20 µm, and the camera is an Andor iXon 860 EMCCD with an array size of 128×128, pixel size 24 µm. (B) Spectral curve, generated by the sum projection of (A) along the *x* dimension; the peak wavelengths are identified from the specification sheet of the calibration lamp. The blue, green, and red lines correspond to the regions in (A) with the same color. All three colored lines almost overlap with each other, which demonstrates a very small distortion in the spectral lines. (C) Dispersion curve, generated from the spectral curve (C), by polynomial fit of specific wavelengths.
(PNG)

Figure S6 **4D PSF theoretical.** The 4D PSF is calculated from the theory as described in **Text S3**. The spectra and wide field point spread function is based on 100 nm yellow bead. The simulation includes aberrations generated from phase retrieving process. A linear stretch (A) and log stretch (B) of intensity for projections on the *x*–*y*, *x*–*z*, and *y*–*z* planes are shown along with the *x*, *y*, and *z* vs. wavelength projections (C, D, and E).
(PNG)

Figure S7 **4D PSF experimental.** The sample is a 100 nm TetraSpeck bead imaged at 100 nm steps in z. A linear stretch (A) and log stretch (B) of intensity for projections on the *x*–*y*, *x*–*z*, and *y*–*z* planes are shown along with the *x*, *y*, and *z* vs. wavelength projections (C, D, and E). The figures show three emission peaks of TetraSpeck bead, 505/515 nm (green), 560/580 nm (orange) and 660/680 nm (dark red). Data were acquired at room temperature, using a 60× water objective, *NA* = 1.2.
(PNG)

Figure S8 **Color dependence of localizations.** Scatter plot of 300 localizations of a single 100 nm TetraSpeck bead on glass using three different wavelength ranges. The purpose here is to quantify any chromatic dependence on particle localization. Emitters were 100 nm Tetraspeck beads diluted 2000∶1 in 1× PBS and allowed to stick to a glass cover slip. The sample was then scanned 300 times and localizations were obtained for each scan using emitted light from each of three different wavelength ranges. The mean of the localized *x*-positions varied by ∼10 nm and the mean of the localized *y*-positions varied by ∼8 nm. (A) Scatter plot of localizations with the mean identified by ‘+’ and listed at the top of the figure. (B) Error bars indicating one standard deviation.
(PNG)

Figure S9 **Hyperspectral viewing GUI.** Hyperspectral viewing GUI provides real-time feedback for instrument control (focusing, ROI determination, etc.) and experimental observations (sample quality, QD labeling density, etc.). The GUI can also be used for preliminary investigations of data in post processing. For example, the blue, green, and red boxes in the RGB image (top left of GUI) highlight single 565, 625, and 800 nm QDs respectively. Spectra for pixels within those boxes are shown in the axes below the RGB image. The GUI provides flexibility in the coloration scheme for the RGB image. For the default RGB representation, each spectral channel is assigned an RGB color progressing from blue to green to red. RGB values for each spatial pixel are determined by relative contributions from of RGB values from each spectral channel.
(PNG)

Figure S10 **3D Gaussian model.** (A) 3D pixelated Gaussian model for a single QD with Poisson noise. (B) The standard deviation in parameter estimates from 500 fits of simulated data with Poisson noise (open circles) agree well with the theoretical standard deviation (square root of the Cramér Rao Bound; *x*). No blinking was included in this simulation. (C) and (D) show results for fitting. Simulations of 2 QDs with a fixed spatial separation (0.5 pixels or ∼60 nm) and variable spectral separation (0 to 50 nm) are fit with a 2 QD model. (C) The relative accuracies (absolute error (ε) normalized by the pixel size) for positional parameters for QD 1 (solid lines; *θ_x1_*, *θ_y1_*, *θ_λ1_*) and QD 2 (dashed lines; *θ_x2_*, *θ_y2_*, *θ_λ2_*). (D) The percent of divergent fits (yielded unreasonable parameter estimates).
(PNG)

Figure S11 **Multi-QD fitting routine.** QD fitting is performed sequentially for 1 to *N* QDs. Figure adapted from Huang et al. More details about the fitting steps are included in **Text S5**.
(PNG)

Figure S12 **QD characterization (525–800).** The rows contain figures related to the indicated QD species. Column 1 shows spectral features of individual QDs (colored lines; 5 randomly selected spectra) and the ensemble of all individual QDs (black line; total number of spectra used to compute each ensemble is included in **Table S2**). Column 2 contains histograms of fit spectral emission peaks (*θ_λ_*) identified by 3D Gaussian fitting (*x*,*y*,*λ*) for individual QDs. Red ticks indicate the expected emission peak for each QD specie. Note that the expected emission peak for QD800 is significantly different from the histogram. This is attributed to the spectral range of the camera (i.e. only the tail of the spectrum is observed on the camera). Column 3 contains histograms of the fit standard deviation in the spectral dimension (*θ_σλ_*) identified by 3D Gaussian fitting for individual QDs. Column 4 contains histograms of fit photons per frame (*θ_σλ_*) for individual QDs. **Table S1** includes further description of the model parameters. Statistics are included in **Table S2**.
(PNG)

Figure S13 **QD localization and blinking.** (A) Localizations for a single QD605 overlaid on the sum projection of raw data. Coloration of localizations corresponds to the localized emission peak (color bar). The estimated intensity (left axis; blue) and spectral emission peak (right axis; green) are shown over time (∼3.8 fps) in (B). Note the blue shifting of the QD is observed in (A) and (B). Localizations were binned by intensity in (C) and the standard deviation of the localization parameters were compared to the square root of the CRB in (D), (E), and (F). The standard deviation in the line dimension (E) corresponds well to the theoretical lower bound. The standard deviation in the scanning dimension (D) and the spectral dimension (F) do not correspond well with theory. This is attributed to QD blinking during the line scan and QD blue shifting respectively.
(PNG)

Figure S14 **Spectral information improves trajectory connections.** Hyperspectral image data (100 frames) with diffusing QDs were simulated (QD simulation parameters attained from the characterization of QDs in **Figure S12** and **Table S2**) and localized (**Text S4**, **Figure S10**, **Text S5**, and **Figure S11**). Trajectories were subsequently built either utilizing (solid colored lines) or ignoring spectral information (solid black lines). Localizations are shown in ellipsoids (size of the ellipsoid corresponds to 3× the estimated localization accuracy), and solid colored lines correspond to the connected trajectories. Dashed colored lines represent the ‘true’ simulated trajectories. The black line with circles identifies trajectories built from connecting localizations without spectral information. Black diamonds represent positions were tracks jump from one true underlying track to another.
(PNG)

Figure S15 **3D scanning of live cells.** Change in cell morphology upon activation of IgE-FcεRI by crosslinking with multivalent antigen (DNP-BSA). (A), (B), and (C) show 3D isosurface representations of deconvolved z stacks for a single cell acquired at 1 hyperspectral image stack every 7.72 seconds at several time points before and after activation as indicated. Cells are labeled with Cell Mask orange (blue), Actin-GFP (green), and QD-IgE-FcεRI (red). See **Video S6**.
(PNG)

Text S1 **Details about general instrument design.**
(DOCX)

Text S2 **Principles of spectrometer design.**
(DOCX)

Text S3 **Theoretical point spread function of HSM.**
(DOCX)

Text S4 **3D Gaussian fitting.**
(DOCX)

Text S5 **Additional details about multi-QD fitting routine.**
(DOCX)

Text S6 **Building trajectories from localizations.**
(DOCX)

Text S7 **Diffusion coefficient uncertainty.**
(DOCX)

Text S8 **Theoretical performance of line and point scanning systems.**
(DOCX)

Table S1 Description of Gaussian fitting variables.
(DOCX)

Table S2 Single QD characterization.
(DOCX)
