# Supplementary figures and images for: Multi-Color Quantum Dot Tracking Using a High-Speed Hyperspectral Line-Scanning Microscope
Source: PLoS One. 2013 May 22;8(5):e64320. doi: 10.1371/journal.pone.0064320 (PMC3661486; doi:10.1371/journal.pone.0064320)

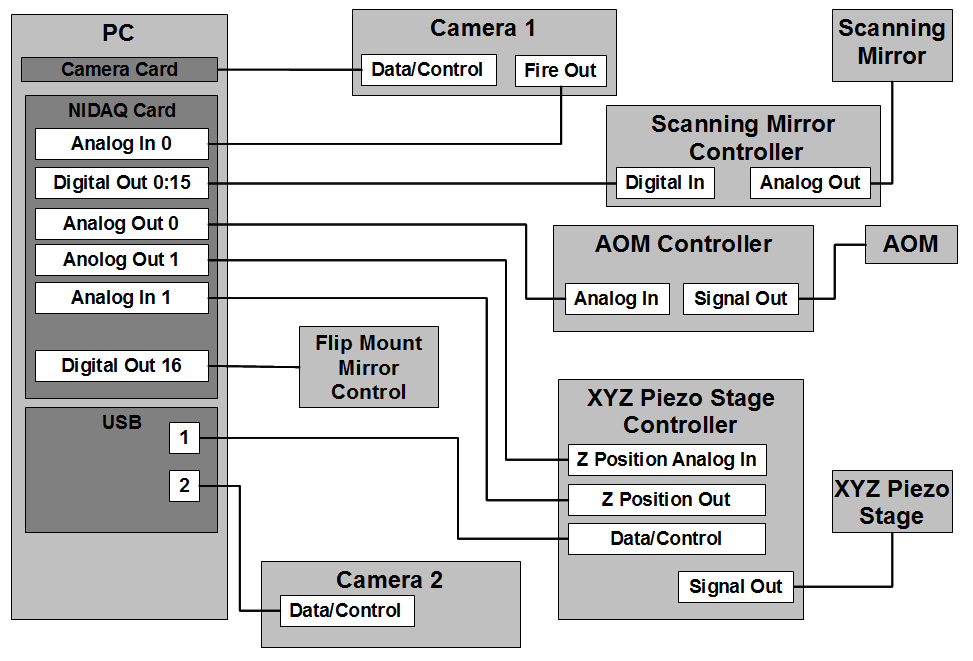

Supplement: Figure S1 — Instrument control schematic. The fire out signal from the camera is used as the clock to advance the scanning mirror, stepping through a list of digital words provided to the scanning mirror control board. The acousto-optic modulator (AOM) controller accepts analog DC voltage input and controls the RF power output to the AOM's piezoelectric transducer. The flip-mount controller toggles the mirror position with a single 5V pulse. The XYZ Piezo-stage controller applies a z-position change based on analog DC voltage input and returns position information to an analog voltage input on the data acquisition board. The secondary camera (used for widefield viewing to center a sample or select ROI) uses a single USB connection. (PNG) [file pone.0064320.s007.png]

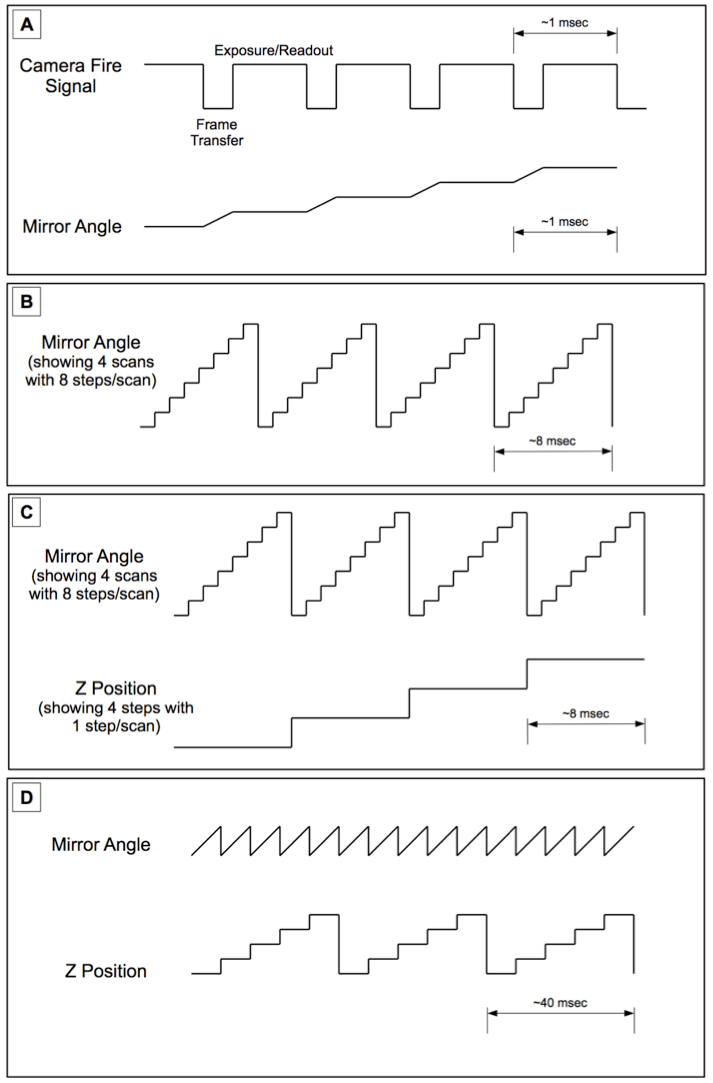

Supplement: Figure S2 — Signal timing. (A) As the camera finishes an exposure the falling edge trigger is used as a clock to advance the mirror scan angle by one step. A frame transfer mode EMCCD camera is used, so readout occurs during exposure. The mirror step response time actually exceeds the frame transfer interval, but is much less than a single exposure, such that mirror motion is negligible during acquisition. (B) Scans are completed after a set number of steps, where the mirror angle is returned to the starting position. (C) In fast 3D scans, as a single scan is completed the z stage is translated by one step. (D) As the z steps are completed through the depth of the region of interest, the z stage is returned to the starting position to begin another pass. (PNG) [file pone.0064320.s008.png]

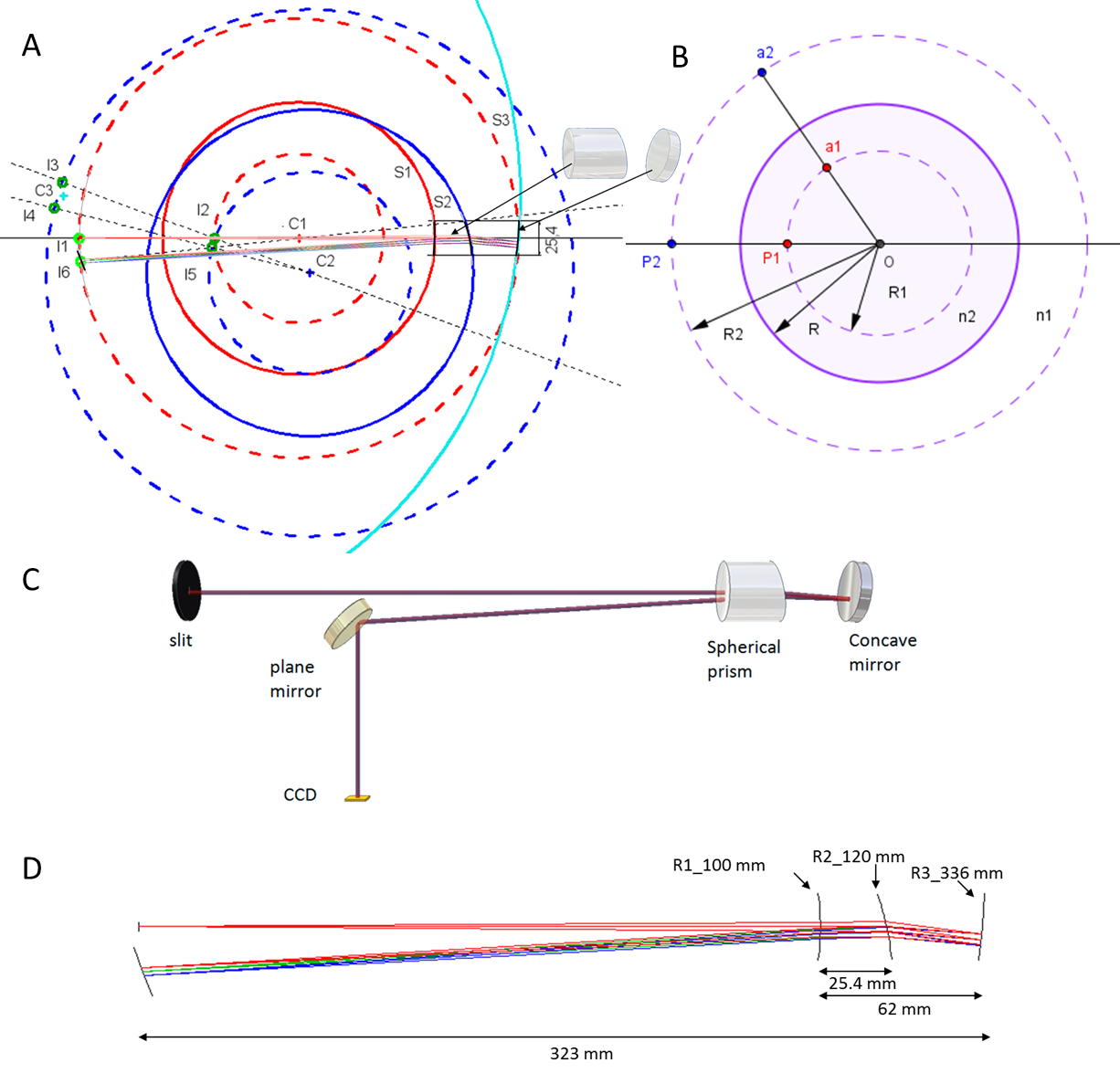

Supplement: Figure S3 — Spectrometer design. A more detailed discussion of the principles of the spectrometer design is in Text S2. (A) Relative positions of each spherical surface of the prism spectrometer. S1 (red solid circle) and S2 (blue solid circle) are refractive surfaces of the spherical prism, S3 (cyan solid circle) is the reflective surface of the concave mirror. The dotted circles are conjugate aplanatic surfaces of S1 and S2. (B) Relationship of conjugate aplanatic surfaces (dotted circles) of the refractive spherical surfaces (solid circles). (C) 3D drawing of the spectrometer. (D) Spectrometer design in OSLO (Optics Software for Layout and Optimization). (PNG) [file pone.0064320.s009.png]

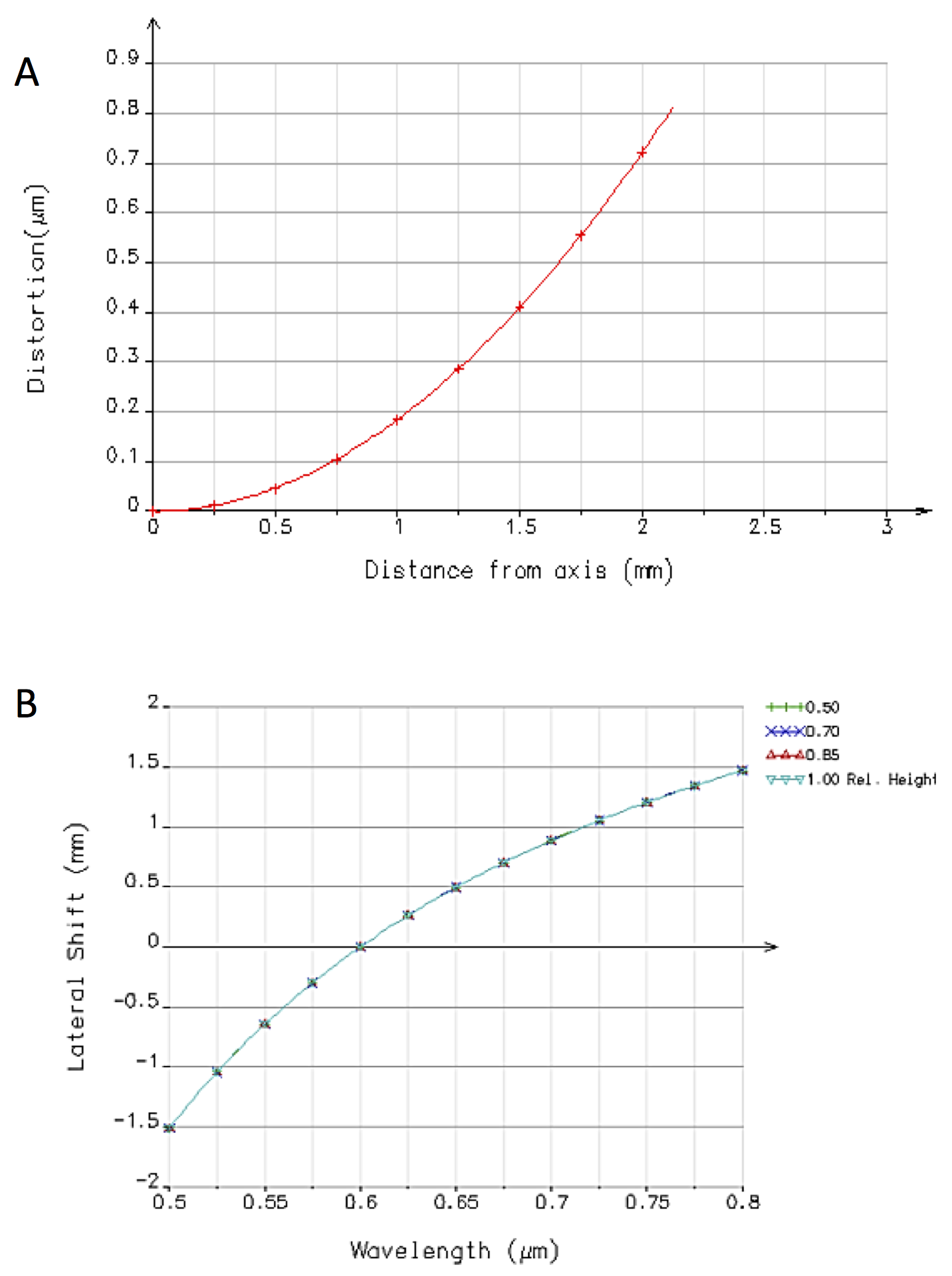

Supplement: Figure S4 — Spectral distortion and dispersion. The distortion and dispersion curves are generated from OSLO. (A) Distortion shows offset of the true image position from the ideal image position at various off axis points. In the HSM setup, the maximum distance from the axis is 1.5 mm, so the designed maximum distortion is 0.4 µm, which is less than 2% of CCD pixel size (24 µm). (B) Lateral shift shows the dispersion, designed to span a total of 3 mm from 500 nm to 800 nm. It shows dispersions in four relative heights (distance from the optical axis), the four curves are right on top of each other, so the spectral lines are straight, which also indicates a minimal distortion. (PNG) [file pone.0064320.s010.png]

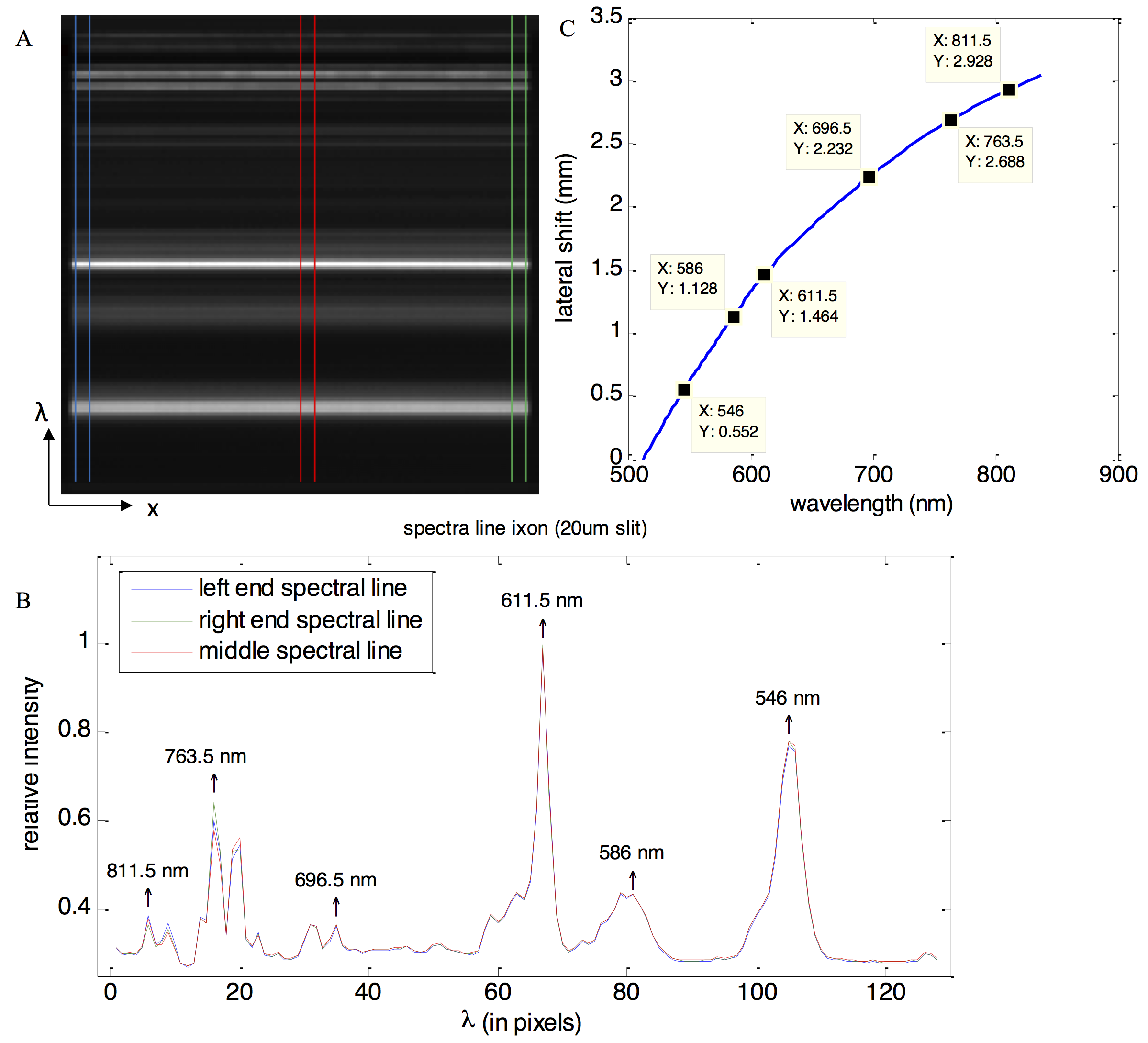

Supplement: Figure S5 — Spectral calibration. (A) Image of the calibration lamp on the camera (the vertical axis is the spectral dimension). The calibration lamp is a LightForm multi-ion discharge lamp (MIDL), the slit width is 20 µm, and the camera is an Andor iXon 860 EMCCD with an array size of 128×128, pixel size 24 µm. (B) Spectral curve, generated by the sum projection of (A) along the x dimension; the peak wavelengths are identified from the specification sheet of the calibration lamp. The blue, green, and red lines correspond to the regions in (A) with the same color. All three colored lines almost overlap with each other, which demonstrates a very small distortion in the spectral lines. (C) Dispersion curve, generated from the spectral curve (C), by polynomial fit of specific wavelengths. (PNG) [file pone.0064320.s011.png]

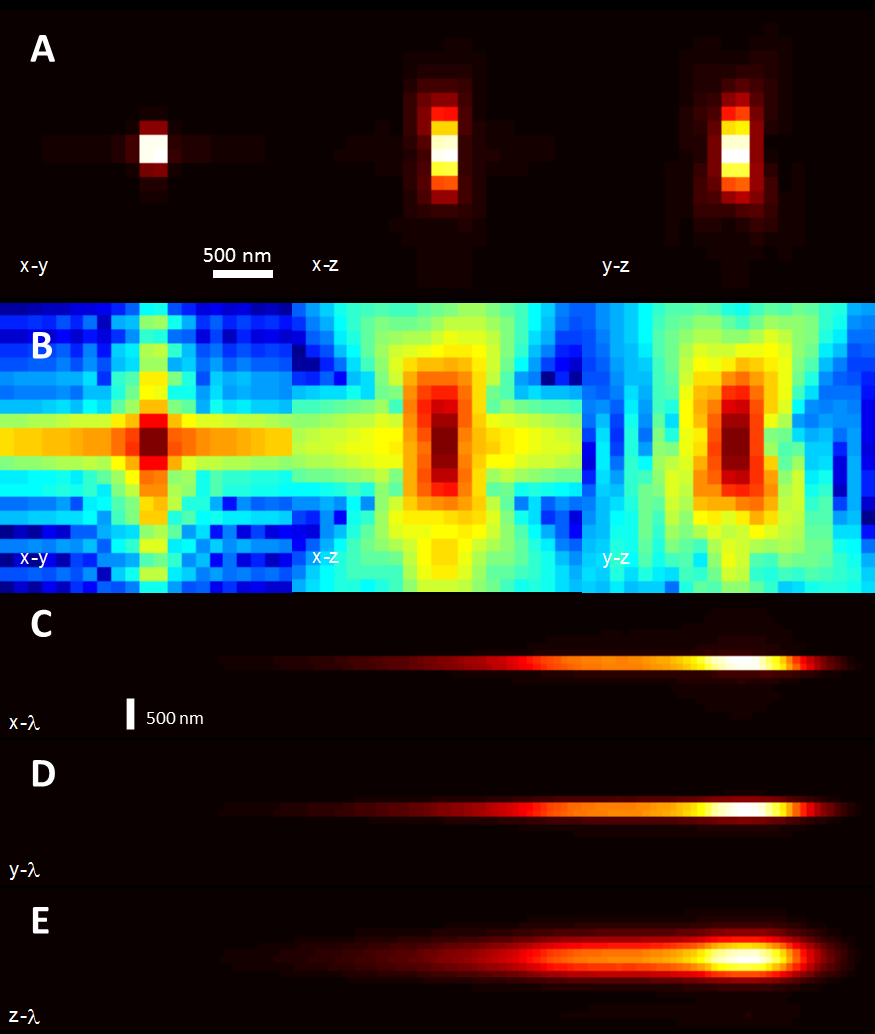

Supplement: Figure S6 — 4D PSF theoretical. The 4D PSF is calculated from the theory as described in Text S3. The spectra and wide field point spread function is based on 100 nm yellow bead. The simulation includes aberrations generated from phase retrieving process. A linear stretch (A) and log stretch (B) of intensity for projections on the x–y, x–z, and y–z planes are shown along with the x, y, and z vs. wavelength projections (C, D, and E). (PNG) [file pone.0064320.s012.png]

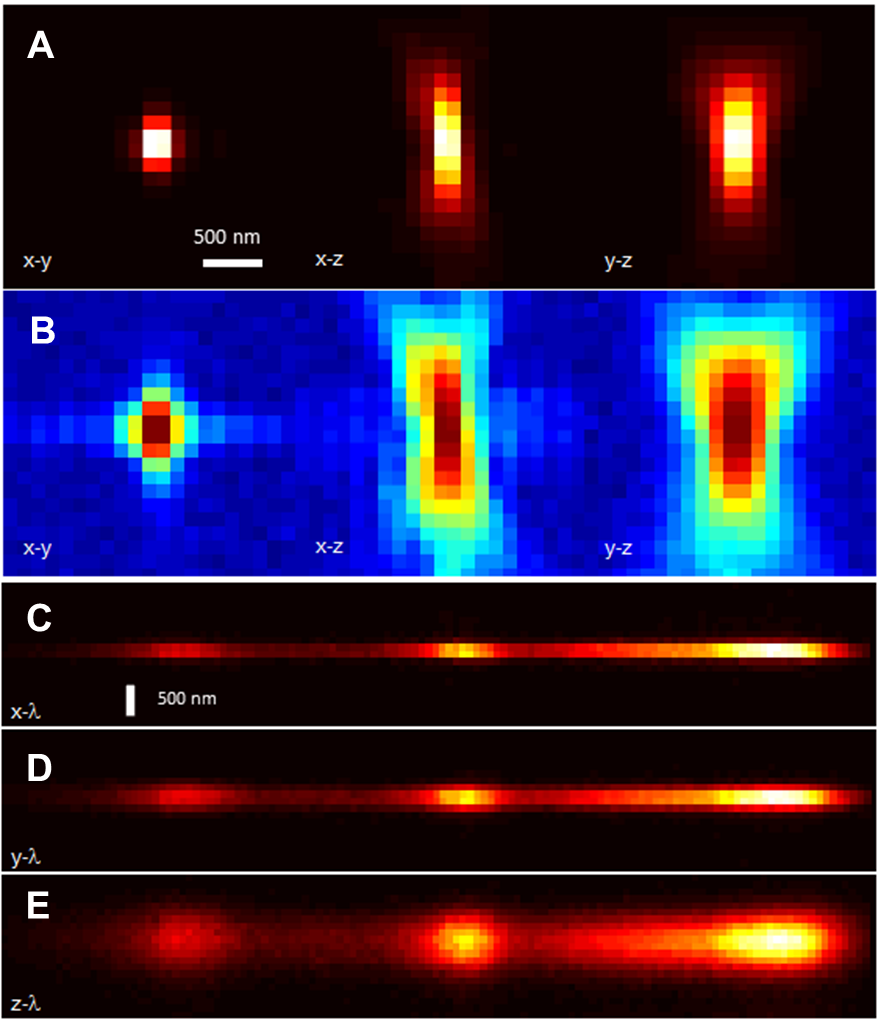

Supplement: Figure S7 — 4D PSF experimental. The sample is a 100 nm TetraSpeck bead imaged at 100 nm steps in z. A linear stretch (A) and log stretch (B) of intensity for projections on the x–y, x–z, and y–z planes are shown along with the x, y, and z vs. wavelength projections (C, D, and E). The figures show three emission peaks of TetraSpeck bead, 505/515 nm (green), 560/580 nm (orange) and 660/680 nm (dark red). Data were acquired at room temperature, using a 60× water objective, NA = 1.2. (PNG) [file pone.0064320.s013.png]

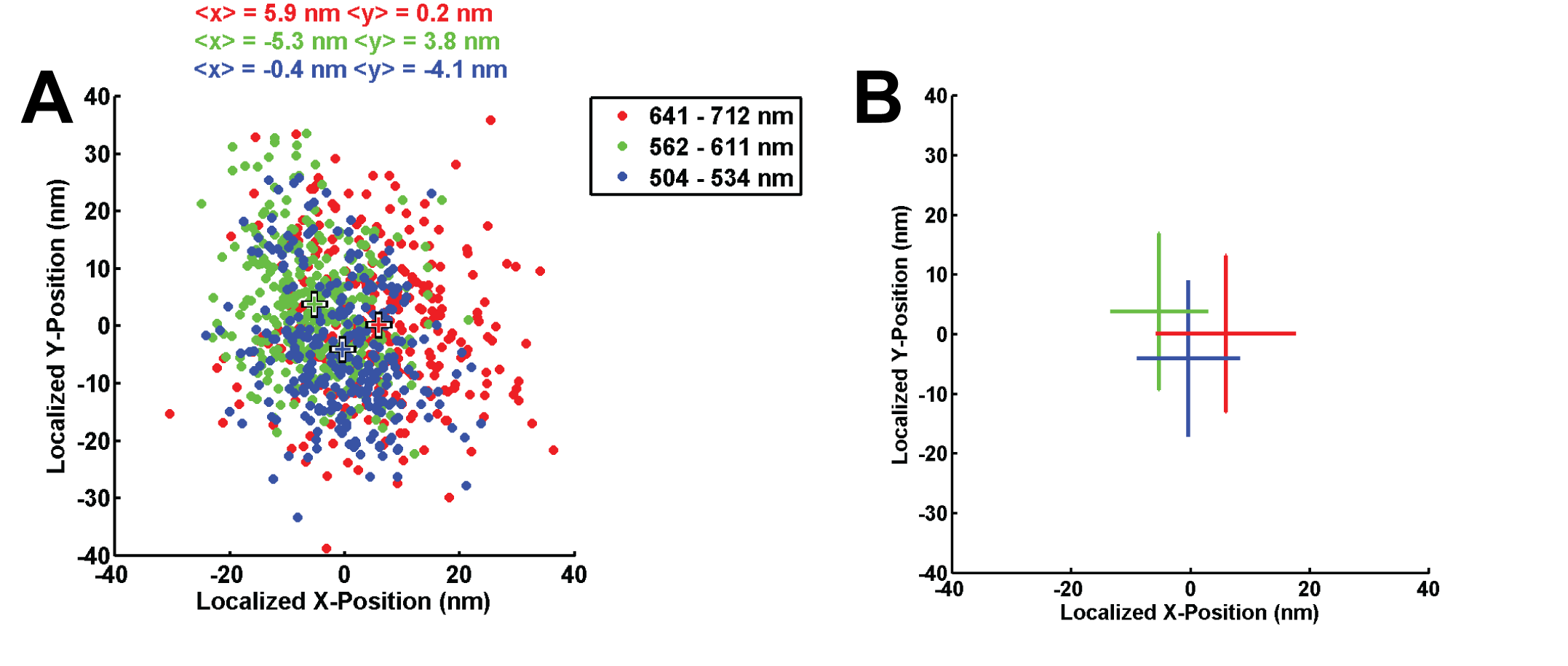

Supplement: Figure S8 — Color dependence of localizations. Scatter plot of 300 localizations of a single 100 nm TetraSpeck bead on glass using three different wavelength ranges. The purpose here is to quantify any chromatic dependence on particle localization. Emitters were 100 nm Tetraspeck beads diluted 2000∶1 in 1× PBS and allowed to stick to a glass cover slip. The sample was then scanned 300 times and localizations were obtained for each scan using emitted light from each of three different wavelength ranges. The mean of the localized x-positions varied by ∼10 nm and the mean of the localized y-positions varied by ∼8 nm. (A) Scatter plot of localizations with the mean identified by ‘+’ and listed at the top of the figure. (B) Error bars indicating one standard deviation. (PNG) [file pone.0064320.s014.png]

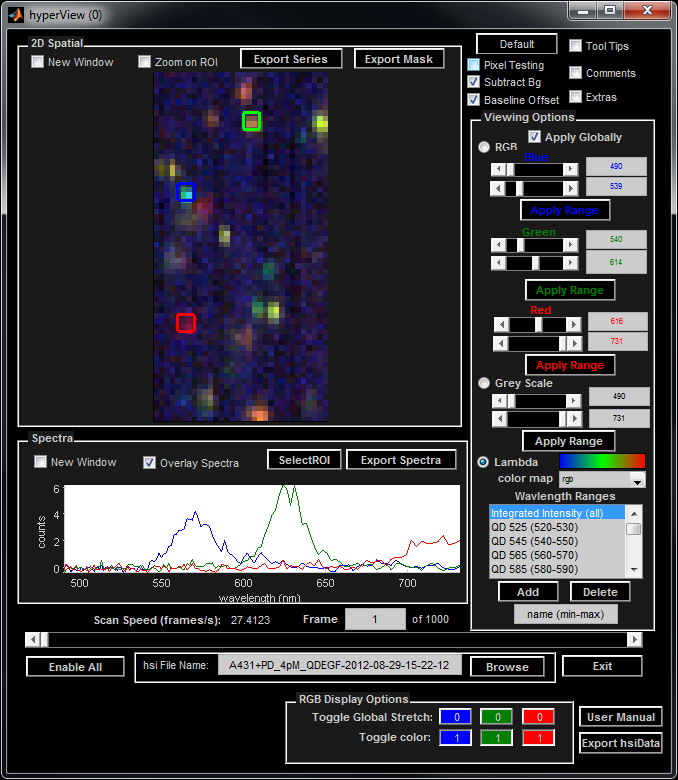

Supplement: Figure S9 — Hyperspectral viewing GUI. Hyperspectral viewing GUI provides real-time feedback for instrument control (focusing, ROI determination, etc.) and experimental observations (sample quality, QD labeling density, etc.). The GUI can also be used for preliminary investigations of data in post processing. For example, the blue, green, and red boxes in the RGB image (top left of GUI) highlight single 565, 625, and 800 nm QDs respectively. Spectra for pixels within those boxes are shown in the axes below the RGB image. The GUI provides flexibility in the coloration scheme for the RGB image. For the default RGB representation, each spectral channel is assigned an RGB color progressing from blue to green to red. RGB values for each spatial pixel are determined by relative contributions from of RGB values from each spectral channel. (PNG) [file pone.0064320.s015.png]

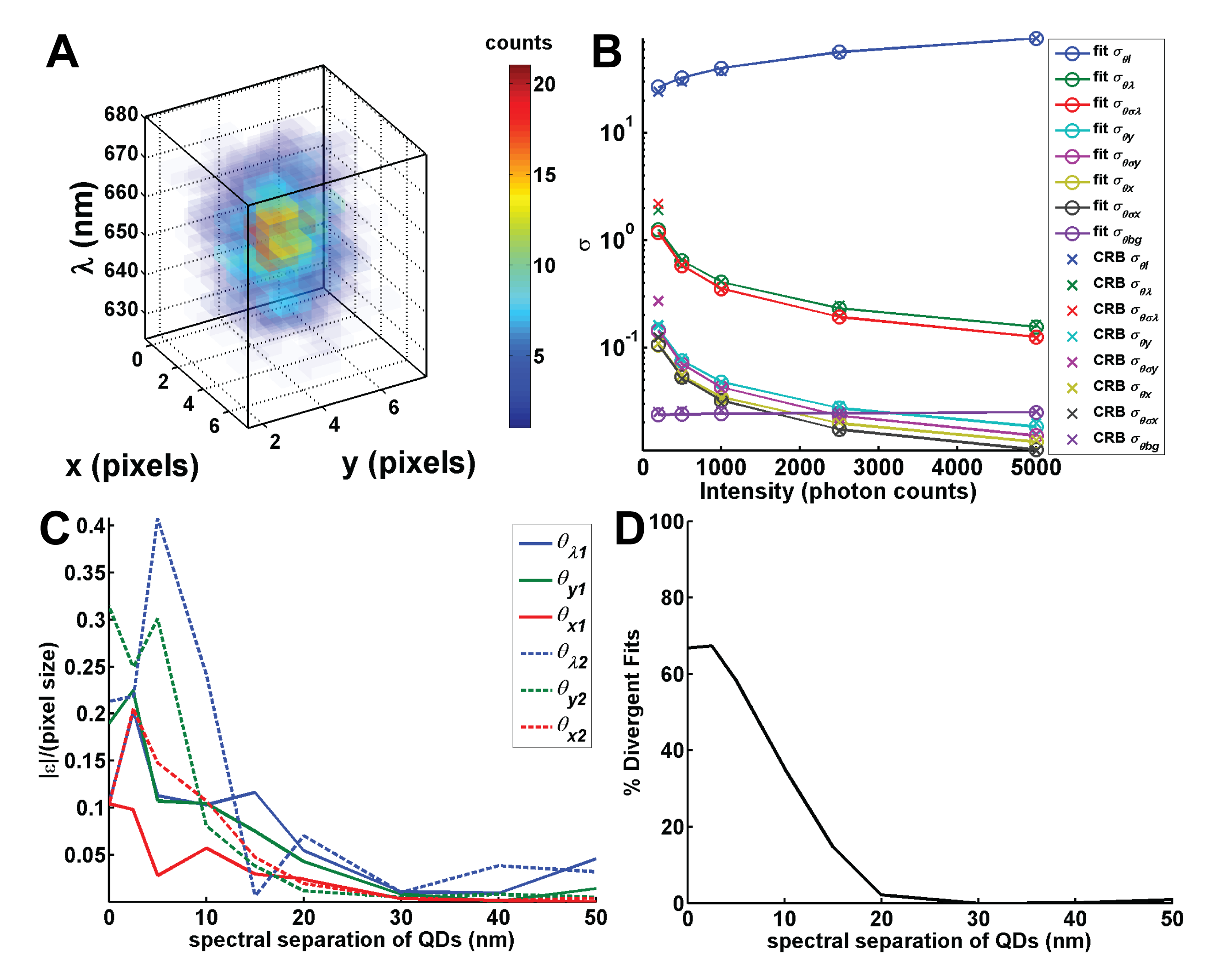

Supplement: Figure S10 — 3D Gaussian model. (A) 3D pixelated Gaussian model for a single QD with Poisson noise. (B) The standard deviation in parameter estimates from 500 fits of simulated data with Poisson noise (open circles) agree well with the theoretical standard deviation (square root of the Cramér Rao Bound; x). No blinking was included in this simulation. (C) and (D) show results for fitting. Simulations of 2 QDs with a fixed spatial separation (0.5 pixels or ∼60 nm) and variable spectral separation (0 to 50 nm) are fit with a 2 QD model. (C) The relative accuracies (absolute error (ε) normalized by the pixel size) for positional parameters for QD 1 (solid lines; θx1, θy1, θλ1) and QD 2 (dashed lines; θx2, θy2, θλ2). (D) The percent of divergent fits (yielded unreasonable parameter estimates). (PNG) [file pone.0064320.s016.png]

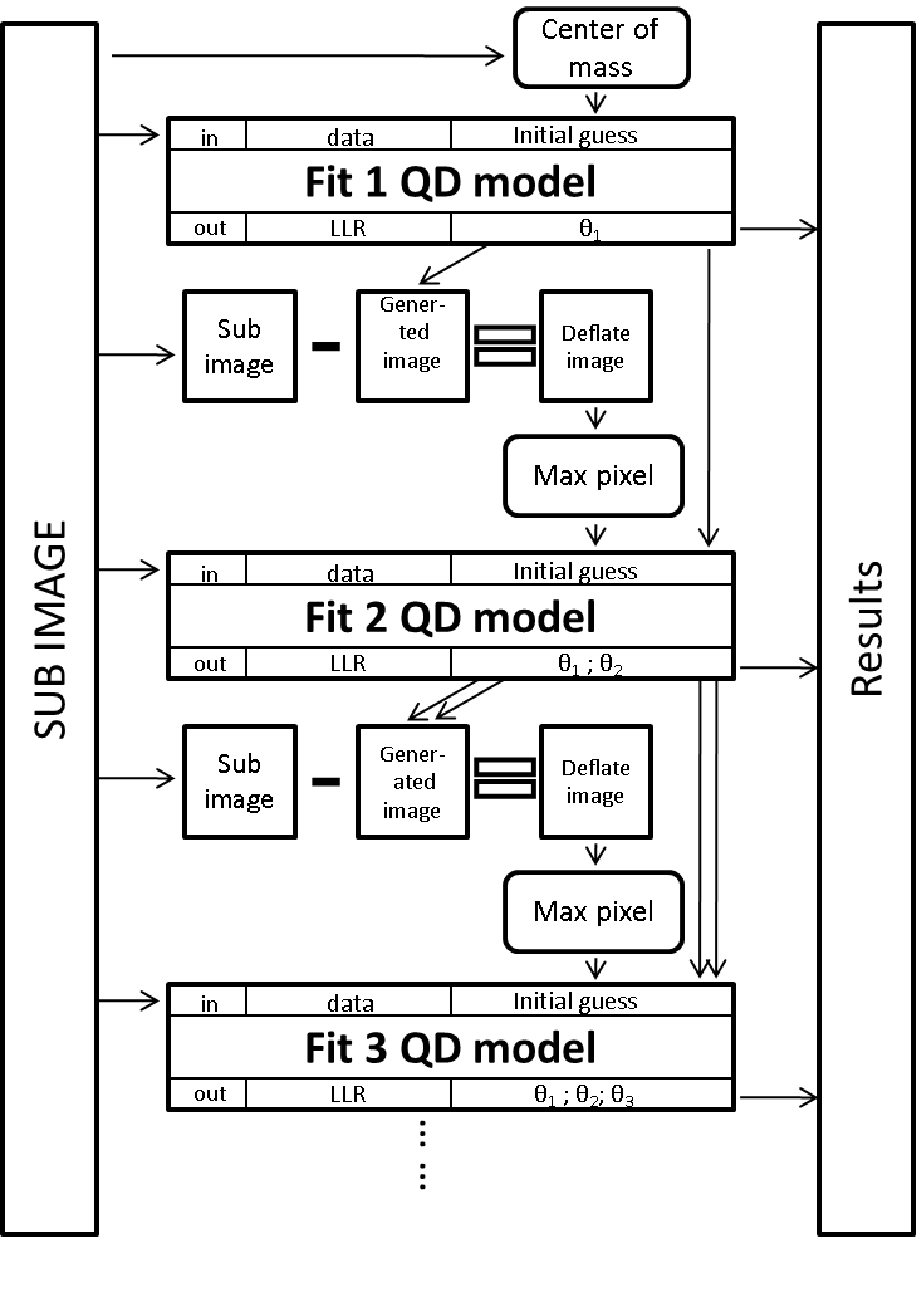

Supplement: Figure S11 — Multi-QD fitting routine. QD fitting is performed sequentially for 1 to N QDs. Figure adapted from Huang et al. More details about the fitting steps are included in Text S5. (PNG) [file pone.0064320.s017.png]

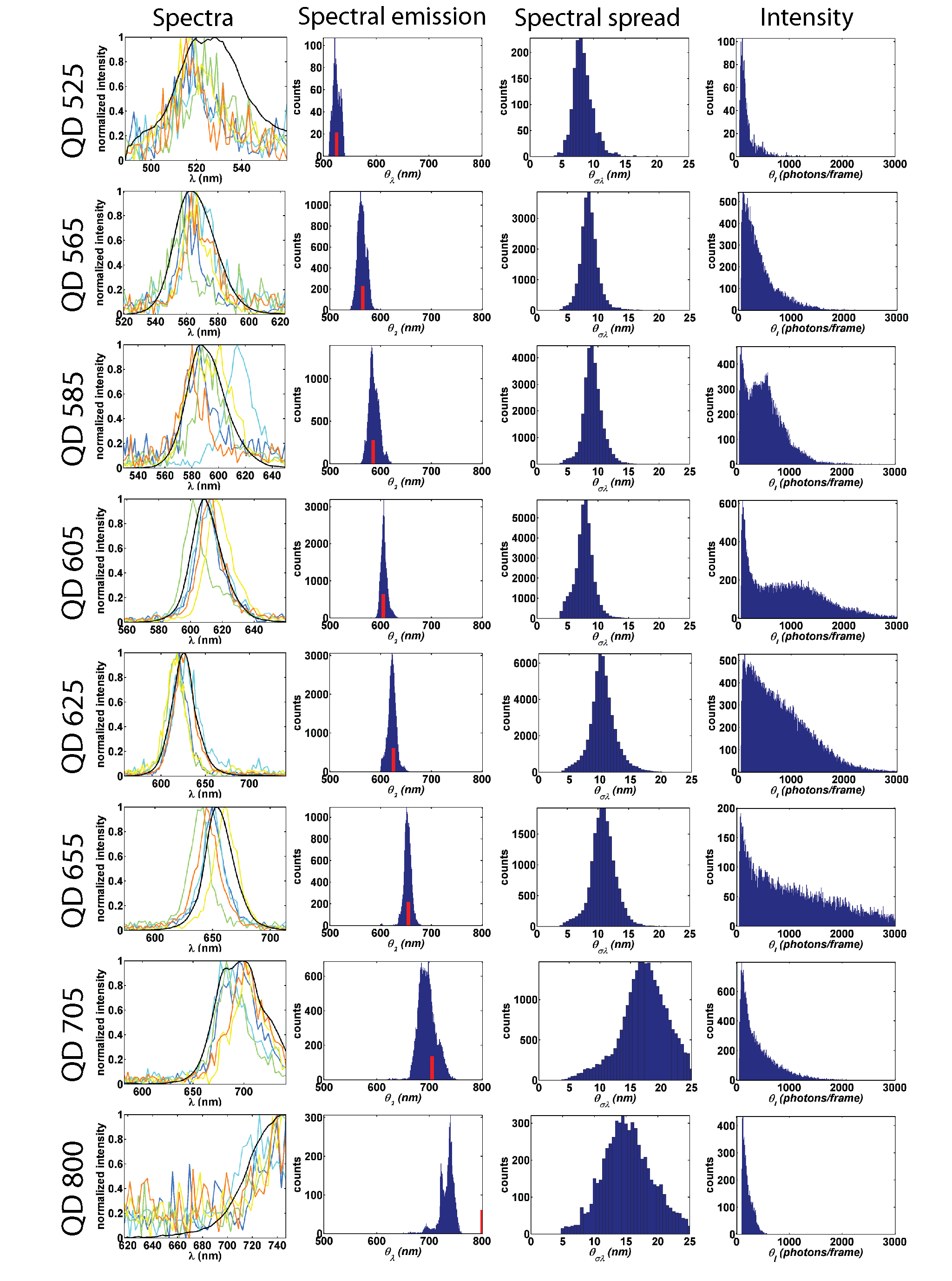

Supplement: Figure S12 — QD characterization (525–800). The rows contain figures related to the indicated QD species. Column 1 shows spectral features of individual QDs (colored lines; 5 randomly selected spectra) and the ensemble of all individual QDs (black line; total number of spectra used to compute each ensemble is included in Table S2). Column 2 contains histograms of fit spectral emission peaks (θλ) identified by 3D Gaussian fitting (x,y,λ) for individual QDs. Red ticks indicate the expected emission peak for each QD specie. Note that the expected emission peak for QD800 is significantly different from the histogram. This is attributed to the spectral range of the camera (i.e. only the tail of the spectrum is observed on the camera). Column 3 contains histograms of the fit standard deviation in the spectral dimension (θσλ) identified by 3D Gaussian fitting for individual QDs. Column 4 contains histograms of fit photons per frame (θσλ) for individual QDs. Table S1 includes further description of the model parameters. Statistics are included in Table S2. (PNG) [file pone.0064320.s018.png]

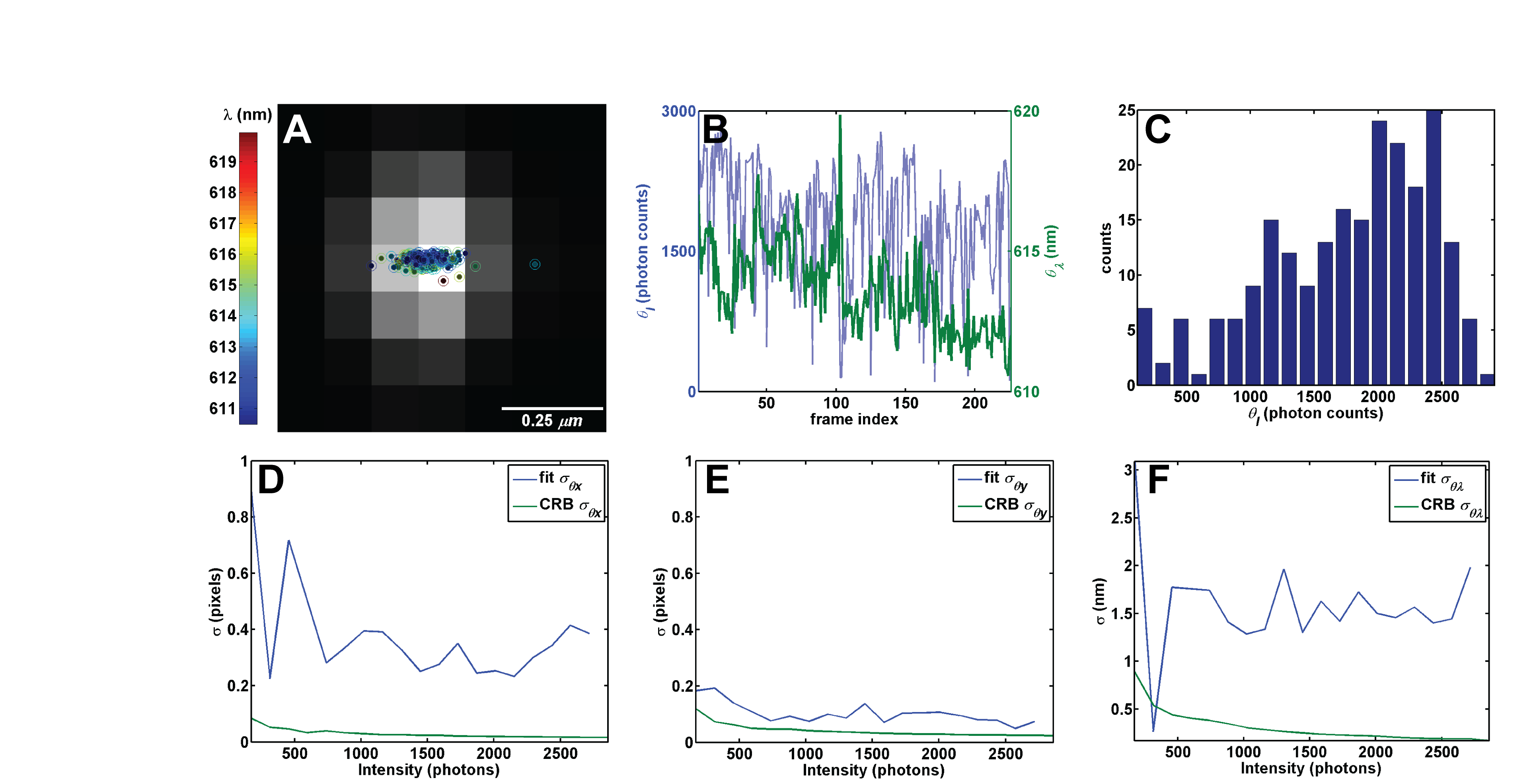

Supplement: Figure S13 — QD localization and blinking. (A) Localizations for a single QD605 overlaid on the sum projection of raw data. Coloration of localizations corresponds to the localized emission peak (color bar). The estimated intensity (left axis; blue) and spectral emission peak (right axis; green) are shown over time (∼3.8 fps) in (B). Note the blue shifting of the QD is observed in (A) and (B). Localizations were binned by intensity in (C) and the standard deviation of the localization parameters were compared to the square root of the CRB in (D), (E), and (F). The standard deviation in the line dimension (E) corresponds well to the theoretical lower bound. The standard deviation in the scanning dimension (D) and the spectral dimension (F) do not correspond well with theory. This is attributed to QD blinking during the line scan and QD blue shifting respectively. (PNG) [file pone.0064320.s019.png]

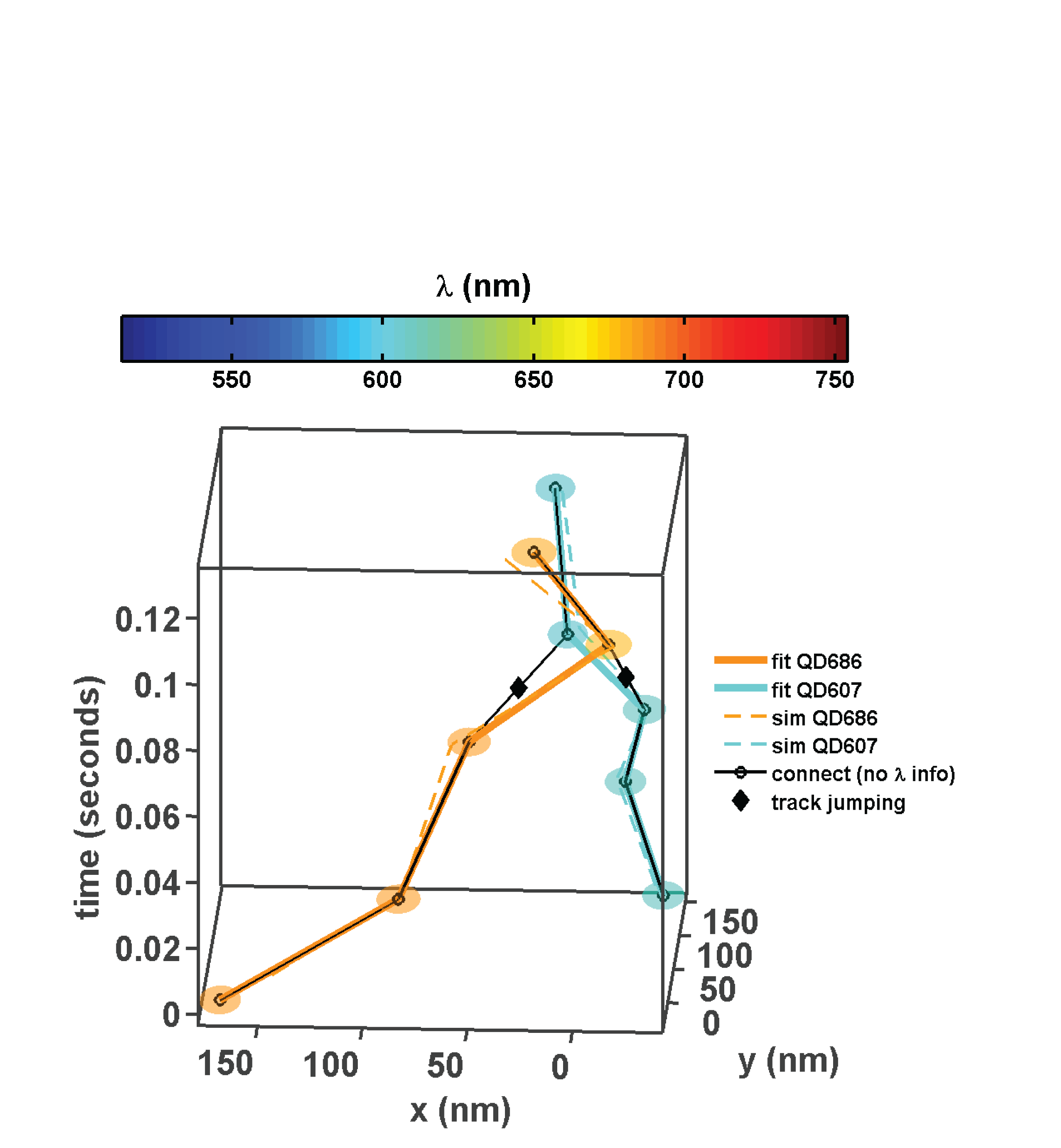

Supplement: Figure S14 — Spectral information improves trajectory connections. Hyperspectral image data (100 frames) with diffusing QDs were simulated (QD simulation parameters attained from the characterization of QDs in Figure S12 and Table S2) and localized (Text S4, Figure S10, Text S5, and Figure S11). Trajectories were subsequently built either utilizing (solid colored lines) or ignoring spectral information (solid black lines). Localizations are shown in ellipsoids (size of the ellipsoid corresponds to 3× the estimated localization accuracy), and solid colored lines correspond to the connected trajectories. Dashed colored lines represent the ‘true’ simulated trajectories. The black line with circles identifies trajectories built from connecting localizations without spectral information. Black diamonds represent positions were tracks jump from one true underlying track to another. (PNG) [file pone.0064320.s020.png]

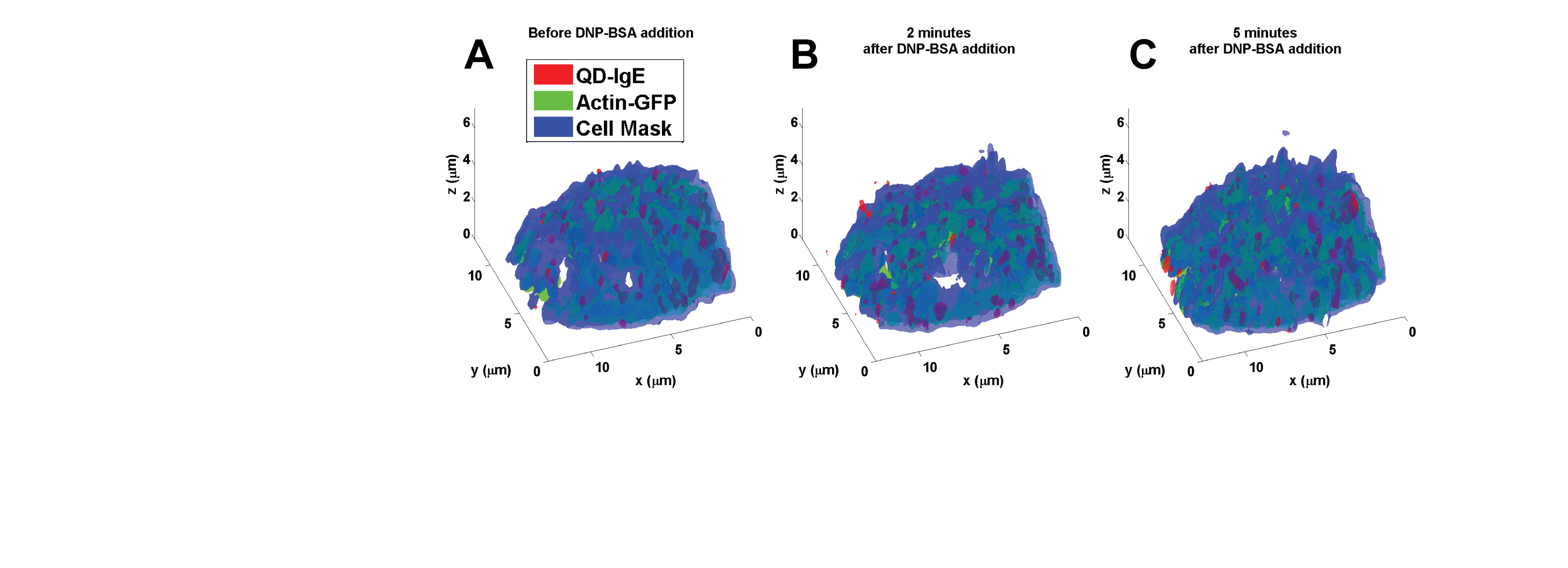

Supplement: Figure S15 — 3D scanning of live cells. Change in cell morphology upon activation of IgE-FcεRI by crosslinking with multivalent antigen (DNP-BSA). (A), (B), and (C) show 3D isosurface representations of deconvolved z stacks for a single cell acquired at 1 hyperspectral image stack every 7.72 seconds at several time points before and after activation as indicated. Cells are labeled with Cell Mask orange (blue), Actin-GFP (green), and QD-IgE-FcεRI (red). See Video S6. (PNG) [file pone.0064320.s021.png]
