# Supplementary material for: Multi-Color Quantum Dot Tracking Using a High-Speed Hyperspectral Line-Scanning Microscope
Source: PLoS One. 2013 May 22;8(5):e64320. doi: 10.1371/journal.pone.0064320 (PMC3661486; doi:10.1371/journal.pone.0064320)
Supplement: Text S1 — Details about general instrument design. (DOCX) [file pone.0064320.s022.docx]

**Text S1. Details About General Instrument Design.**

Unless otherwise specified, the spatial dimensions will be defined such that the *z*-axis is along beam line, the *y*-axis will be perpendicular to the optics table surface, and the *x*-axis will be parallel to the optics table surface. Beam steering mirrors, optics mounts, and micro-positioners are omitted to simplify the diagram. The numbers in brackets {} are references to parts listed below.

## Excitation Path:

Starting with the 488 nm laser (**Figure 2** upper left, {1}) the beam is 40 mW which turns out to be much more than needed for most experiments with this system, so neutral density filters ({2}, {3}) are used for coarse adjustment of the initial laser power. The beam is steered (using mirrors {4}, {5}) through an acousto-optic modulator (AOM, {6}) and the first order diffracted beam is coupled into the fiber {7}, {8}. The amount of laser light in the coupled beam depends on the radio frequency (RF) power in the AOM, which can be smoothly and quickly controlled, providing a good mechanism for modulation and tuning the power of the laser light as desired. The AOM power is controlled by providing a voltage to the signal processor unit. This voltage signal is provided by an analog output from a data acquisition and control PC card {9} that is controlled via software. The fiber is single-mode, polarization preserving and is used as a means to control intensity as well as for acting as a good spatial filter since light emitted from the fiber is collimated (via objective {10}) into a good approximation of a Gaussian beam TEM 00 mode with an 1/e^2^ diameter of ~0.8 mm. The polarization is maintained such that the light which is focused to create the laser line has the *E* field oriented along the length of the line at the focal plane so that the line has the minimum possible width.

The free space Gaussian beam is steered into a laser line generator lens {11} that has a 15 degree fan angle in the *y*-dimension and distributes the light evenly over the angle. This light is then collimated with a cylindrical lens (CYL1, {12}). Lens CYL2 {13} is a concave cylindrical lens oriented along the *x*-axis, which serves to expand the beam along the *x*-axis such that the beam will ultimately fill the back aperture of the objective lens, which reduces the excitation focal volume along the *z*-axis. Lenses L1 {14} and L2 {17} are used as a telescope to reduce the size of the line before entering the microscope base {18}, such that the line will be ~15 um in length on the sample (which is also the approximate length of one column of 128 CCD pixels back-projected on the sample). L1 focuses the light onto the scanning mirror {16} after being reflected off of the dichroic mirror {15}, which is fixed at a 45-degree angle to the beam.

## Emission Path:

The light emitted from a fluorophore excited by the laser line will be distributed over all angles and the light which is accepted by the aperture of the objective lens {29} will then be collimated and will propagate to the tube lens. The tube lens will focus the light to an image plane which is also the focal plane of lens L2, so it will then be collimated and de-scanned by the mirror, which causes the emission light to match the axis of excitation light for all scan angles, allowing fixed positions of the remaining optical elements.

The emitted light then passes through the dichroic mirror and a 488 nm long pass filter {22}, which has the purpose of further reducing any amount of excitation light that may have passed through the dichroic filter. Lens L3 {23} then focuses this light onto the entrance slit {24} of the spectrometer, which also serves as a line-scanning confocal slit that blocks much of the light from sources that are out of focus.

The custom-made prism spectrometer {27} is described fully in **Text S2**, and focuses the light onto the CCD pixel array (**Figure 2** and **Figure S3**). This provides a 128×128 array of data from the CCD {28} that consists of *y*-position (along the laser line and slit) and wavelength along the other dimension. As the line is scanned across the sample another 128×128 (*y*, *λ*) array is taken for each line position (one angle step in the *x*-dimension). The full field of view (*x*, *y*) is then reconstructed into a data set of (*x*, *y*, and *λ*) for one scan or “hyperspectral image”.

Other (some not shown in diagram): 3D scanning is enabled by the *x*, *y*, *z* piezo stage {19}. Widefield excitation (used for finding regions of interest within a sample) is performed using a white light source {20} and filter cube set {21}. The electromechanical flip-mount mirror {25}, changes the emission path into the widefield CCD {26} and is controlled through the DAQ GUI.

## Parts Reference List

1. Laser: Newport Spectra Physics 488 Cyan, Model PC14011
2. ND Filters: Newport FRQ-ND03, FRQ-ND07, FRQ-ND10, FRQ-ND15, and FRQ-ND20
3. ND Filter Wheels: Newport FWM1X12
4. Mirrors: Newport 10D20ER.1
5. Mirror Mounts: Newport U100-A with precision actuators AJS100-0.5
6. AOM: IntraAction Corp. Model AOM-402AF1 with signal processor from IntraAction Corp. model ME-402
7. Fiber: Newport F-PM480-C-2FC
8. Fiber Coupler: Newport F-91-C1 with objective lens M-40X
9. DAQ Card: National Instruments PCIe-6343 X Series Data Acquisition Card (P/N 781047-01) with BNC-2110 shielded connector block (P/N 777643-01) and SHC68-68-EPM 5m cable (P/N 192061-05).
10. Fiber to Collimated Free Space Beam: Newport L-40X (f = 4.6 mm, NA = 0.66), Thorlabs HPT1 X,Y Translator (holds FC connector), Thorlabs SM1Z Z-Axis Translator (holds the objective)
11. Laser line generator lens: Edmund Optics 46632 (15-degree fan angle)
12. CYL1: Edmund Optics 48353 (f = 25 mm, d = 12.5 mm, MgF2 Coated)
13. CYL2: Thorlabs LK1743R-A (f = -100 mm, d = 25.4 mm, BK7)
14. Lens L1: Newport KPX114AR.14 (f = 350 mm, d = 25.4 mm)
15. Dichroic: Semrock LPD01-488RU-25x36x2.0
16. Scanning Mirror: Cambridge Technology 6210H
17. Lens L2: Newport PAC040AR.14 (f = 50.5 mm, d =25.4 mm)
18. Microscope Base: Olympus IX71
19. X,Y,Z Piezo Stage: Mad City Labs Nano-LPS200
20. Widefield Lamp: X-cite 120Q
21. Widefield Filter Cube Set: Chroma Z473-10x (excitation), Semrock LPD01-488RS-25x36x2.0 (dichroic), Semrock LP02-488RU-25 (emission).
22. Long Pass Filter: Semrock LP02-488RU-25
23. Lens L3: Newport PAC061AR.14 (f = 175 mm, d = 25.4 mm)
24. Spectrometer Entrance Slit: Thorlabs S100R (100 +/- 4 μm wide, 3mm tall)
25. Flip-Mount Mirror: Thorlabs MFF001
26. EMCCD1: Andor Luca R
27. Prism Spectrometer: Custom Design, Rainbow Optics
28. EMCCD2: Andor iXon 860
