# Supplementary material for: Multi-Color Quantum Dot Tracking Using a High-Speed Hyperspectral Line-Scanning Microscope
Source: PLoS One. 2013 May 22;8(5):e64320. doi: 10.1371/journal.pone.0064320 (PMC3661486; doi:10.1371/journal.pone.0064320)
Supplement: Text S2 — Principles of spectrometer design. (DOCX) [file pone.0064320.s023.docx]

## Text S2. Principles of Spectrometer Design.

## Aplanatic image formation of spectrometer

In **Figure S3A**, *S_1_* and *S_2_* are the front and rear spherical surfaces of the spherical prism. *S_3_* is the surface of the concave mirror. *I_1_* is the object point at the entrance slit. *I_6_* is the image point at the spectral plane. *I_2_*-*I_5_* are intermediate image points. *I_1_-I_6_* are on the concentric spherical surfaces of *S_1_* and *S_2_*. Since the concentric surfaces are an aplanatic pair of image and object conjugates, the spectrometer is aplanatic. Those concentric surfaces satisfy the relationship $n_{1}^{2}R_{1}=n_{2}^{2}R_{2}=n_{1}n_{2}R$ in **Figure S3B**.

The detailed image formation of the spectrometer is:

(1) The front surface of prism *S_1_* forms a virtual image of object *I_1_* at the position of its aplanatic conjugates *I_2_*. *C_1_* is the center of surface *S_1_*.

(2) Notice that *I_2_* also lies on the aplanatic conjugate surface of the rear prisms spherical surface *S_2_*, which forms the virtual image of *I_2_* at position *I_3_*. *C_2_* is the center of surface *S_2_*.

(3) Mirror *S_3_* forms a virtual image of *I_3_* at position *I_4_* by reflection. Since *I_3_* and *I_4_* are near the center of *S_3_*, the magnification is 1:1.

(4) Notice that *I_4_* is still at the aplanatic conjugate surface of *S_2_*, which relays *I_4_* to *I_5_*.

(5) Although *I_5_* doesn’t lie on the inner aplanatic conjugate of *S_1_*, it is near the intersection and *S_1_* can still form the real image *I_6_* at the outer aplanatic conjugate of *S_1_*. Therefore, this last refraction is still near aplanatic.

## Calculation of spectrometer design

First, given an approximate size of the spectrometer 300 mm, we choose $R_{1}$=100 mm, and $R_{2}$=120 mm (**Figure S3D**), the glass type of prism is F2, which has a refractive index *n_c_*=1.615 (the refractive index at 656.3 nm). All the calculations follow the aplanatic rule.

$$I_{1}C_{1}=nR_{1}=1.615\times100=161.5 \mathrm{mm}$$

$$I_{2}C_{1}=\frac{R_{1}}{n}=\frac{100}{1.615}=62 \mathrm{mm}$$

$$I_{5}C_{2}=\frac{R_{2}}{n}=\frac{120}{1.615}=74.3 \mathrm{mm}*$$

*It’s 74.56 mm in the final design, so there is a little offset from the perfect image

$$I_{3}C_{2}=nR_{2}=1.615\times120= 193.8 \mathrm{mm}$$

$R_{3}$ is decided by making *I_3_* near *C_3_*. Since *I_4_* should also be on the outer dotted blue circle, *C_3_* should be near the midpoint of *I_3_* and *I_4_*. ${R_{3}=I}_{3}C_{2}+R_{2}+L$, where $L$ is an arbitrary distance. For a starting value, we choose $L=25 \mathrm{mm}$ which gives $R_{3}=338.3 \mathrm{mm}$ ($R_{3}=336 \mathrm{mm}$ in the final design).

These numbers are chosen to be the starting point of the prism spectrometer design in OSLO. The radius and angle of the surfaces *S_2_*, *S_3_*, and the distance between the three surfaces are adjusted to minimize aberrations (mainly astigmatism, coma, and distortion). The table below shows the dimensions of the optimized spectrometer design in OSLO.

Dimensions of each surface in OSLO design

| Surface | Radius | Thickness | Glass | Notes |
| --- | --- | --- | --- | --- |
| object | 0.00 | 261.50 | Air |  |
| 1 | -100.00 | 25.40 | F2 | Tilt 0.00 deg |
| 2 | -120.00 | 36.69 | Air | Position then tilt (*Y*-tilt, clockwise) -12.200 deg |
| 3 | -336.00 | -36.69 | Reflect | Position then tilt (*Y*-tilt, clockwise) 5.458 deg |
| 4 | -120.00 | -25.4 | F2 | Same as *S_2_* |
| 5 | -100.00 | -259.94 | Air | Same as *S_1_* |
| image | 0.00 | 0.00 | Air | Position then tilt (*Y*-tilt, clockwise) -20.879 deg, global coordinate: *Y* is -17.14, *Z* is -259.94, origin is at *S_1_* |

Units are mm.
