# Supplementary material for: Multi-Color Quantum Dot Tracking Using a High-Speed Hyperspectral Line-Scanning Microscope
Source: PLoS One. 2013 May 22;8(5):e64320. doi: 10.1371/journal.pone.0064320 (PMC3661486; doi:10.1371/journal.pone.0064320)
Supplement: Text S3 — Theoretical point spread function of HSM. (DOCX) [file pone.0064320.s024.docx]

## Text S3. Theoretical Point Spread Function of HSM

In the HSM, the excitation path is the same as a line scanning confocal microscope. On the sample plane, assuming that a single fluorophore is at point (0,0,*z*), the excitation laser line is along the *y*-axis at position $x$. The excitation field at the fluorophore is

$U_{\mathrm{ex}}\left( 0-x,z \right)$.

We use $U$ for magnitude point spread function, and $PSF$ for intensity point spread function. $U_{\mathrm{ex}}$ is a line spread function, assuming the intensity along *y*-axis is uniform. In the emission path, after light passes through the slit, the emission field at slit will be dispersed along *x*-axis by spectrometer at the image plane. The field at image plane is generated by shifting the slit field along *x*-axis (spectral dimension) and multiplying each shifted field by the spectral function at the corresponding wavelength. We define the spectral function as $SP(x_{\lambda})$, which depends on the spectrum of the sample. The field at position $(x_{\lambda} , y)$ on image plane is

$\int_{l} U_{\mathrm{em}}\left( x-\xi,y,z \right)L\left( \xi\right)SP(x_{\lambda}+\xi)d\xi$.

$U_{\mathrm{em}}(x,y,z)$ is the wide field emission point spread function, and $L(x)$ is the slit shape function along x axis, which is a rectangular function with the width equal to the slit width $l$ . The above expression is the convolution of $U_{\mathrm{em}}(x,y,z)$ with $L(x)SP(x_{\lambda})$. Combining the excitation and emission fields gives the magnitude point spread function of the HSM

$U_{\mathrm{HSM}}(x,y,z,x_{\lambda})=U_{\mathrm{ex}}\left( -x,z \right)\int_{l} U_{\mathrm{em}}\left( x-\xi,y,z \right)L\left( \xi\right)SP(x_{\lambda}+\xi)d\xi$.

$U_{\mathrm{ex}}\left( -x,z \right)$ is the wide field excitation line spread function. $U_{\mathrm{HSM}}$is a 4D point spread function, with spatial dimension $x,y,z$ , and spectral dimension $x_{\lambda}$. The intensity point spread function is

$PSF_{\mathrm{HSM}}=\left| U_{\mathrm{HSM}} \right|^{2}$.
