# Supplementary material for: Multi-Color Quantum Dot Tracking Using a High-Speed Hyperspectral Line-Scanning Microscope
Source: PLoS One. 2013 May 22;8(5):e64320. doi: 10.1371/journal.pone.0064320 (PMC3661486; doi:10.1371/journal.pone.0064320)
Supplement: Text S4 — 3D Gaussian fitting. (DOCX) [file pone.0064320.s025.docx]

**Text S4. 3D Gaussian Fitting.**

The 3D Gaussian model used to estimate the microscope PSF and spectral features of individual QDs is

$PSF\left( x,y,\lambda\right)=\frac{1}{{(2\pi)}^{\frac{3}{2}}\theta_{\sigma_{x}}\theta_{\sigma_{y}}\theta_{\sigma_{\lambda}}}e^{-\frac{\left( x-\theta_{x} \right)^{2}}{{{2\theta}_{\sigma_{x}}}^{2}}-\frac{\left( y-\theta_{y} \right)^{2}}{{{2\theta}_{\sigma_{y}}}^{2}}-\frac{\left( \lambda-\theta_{\lambda} \right)^{2}}{{{2\theta}_{\sigma_{\lambda}}}^{2}}}$.

Definitions of all variables are included in **Table S1**. Due to the optical setup of the HSM, the intensity for a single QD is distributed over several spatial and spectral pixels. The contribution of a single QD to the detected counts in pixel *k* of an HSM image is modeled by

$\mu_{k}\left( x,y,\lambda\right)=\theta_{I}\int_{A_{k}} PSF\left( u,v,w \right)dudvdw$,

where $A_{k}$ is the area for a single pixel. Integration of the equation above gives

$\mu_{k}\left( x,y,\lambda\right)=\theta_{I}\Delta E_{x}\left( x,y,\lambda\right)\Delta E_{y}\left( x,y,\lambda\right)\Delta E_{\lambda}\left( x,y,\lambda\right)$,

where the contribution of the Gaussian in the spectral dimension is

$\Delta E_{\lambda}\left( x,y,\lambda\right)=\frac{1}{2}\left[ \text{erf}\left( \frac{\lambda-\theta_{\lambda}+\frac{\lambda_{width}}{2}}{\sqrt{2}\theta_{\sigma_{\lambda}}} \right)-\text{erf}\left( \frac{\lambda-\theta_{\lambda}-\frac{\lambda_{width}}{2}}{\sqrt{2}\theta_{\sigma_{\lambda}}} \right) \right]$.

The terms$\Delta E_{x}\left( x,y,\lambda\right)$ and $\Delta E_{y}\left( x,y,\lambda\right)$ are equivalently defined relative to *x* and *y* respectively. The 3D Gaussian model for a single QD is depicted in **Figure S10A**. The model is easily expanded to *N* spatially and spectrally overlapping QDs with background

$\mu_{k_{All}}\left( x,y,\lambda\right)=\sum_{i}^{N} \mu_{k_{i}}\left( x,y,\lambda\right)+\theta_{bg}$.

Model parameters for individual QDs are fit to experimental data by maximizing the likelihood function:

$L\left( \theta|D \right)=\prod_{k} \frac{{\mu_{k_{All}}\left( x,y,\lambda\right)}^{d_{k}}e^{-\mu_{k_{All}}\left( x,y,\lambda\right)}}{d_{k}!}$.

Parameter optimization is performed using the Newton-Raphson method which is an extension of previous work [1]. The variance in parameter estimates approaches the theoretical limit set by the Cramér Rao Bound for simulated data (**Figure S10B**). The accuracy for localizing two highly overlapping emitters simultaneously is demonstrated in **Figure S10C** and **S10D**. Relatively accurate localizations (spatial and spectral) for both QDs is achieved with a high degree of spatial overlap and spectral separation distances down to 20 nm.

Similar to previous work, the 3D Gaussian fitting algorithm is implemented in Compute Unified Device Architecture (CUDA) for Graphics Processing Units (GPUs). The algorithmic implementation for fitting single and multi-QD models for an individual sub image is an adaptation of previous work (**Figure S11**).

QD blinking while the excitation line is scanning over a single QD causes errors in localization in the scan dimension of the line (**Figure S13**). The assumption that the mobility of individual receptors is dimension independent means that this artifact has a minimal impact on physical interpretation of the data. This artifact causes difficulties for the 3D Gaussian model in x (scan dimension) and complicates several facets of SPT in HSM images:

1. The log likelihood ratio test cannot be used to select the appropriate model;
2. The CRB does not accurately approximate the variance in parameter estimates in x (scan dimension).

Thusly, an empirically determined algorithm is used to identify ‘appropriate’ fits (see below).

## Steps for Filtering QD Localizations

1. Eliminate fits with unreasonable fit parameters
2. Eliminate multi-emitter fits in which localizations are too close to one another.
   - An error scaling term ($a$) defined by the user is used to identify closeness of position parameter values. The error scaling term is defined independently for spatial and spectral dimensions.
   - Fits in which position parameters for QD*_i_* ($\theta_{\lambda_{i}}$*,* $\theta_{y_{i}}$ or*,*$\theta_{x_{i}}$) and for QD*_j_* ($\theta_{\lambda_{j}}$*,* $\theta_{y_{j}}$ or*,*$\theta_{x_{j}}$) are closer together than their combined errors multiplied by the error scaling term$a_{\lambda}\sqrt{{\sigma_{\theta_{\lambda_{i}}}}^{2}+{\sigma_{\theta_{\lambda_{j}}}}^{2}}$ , $a_{y}\sqrt{{\sigma_{\theta_{y_{i}}}}^{2}+{\sigma_{\theta_{y_{j}}}}^{2}}$, and $a_{x}\sqrt{{\sigma_{\theta_{x_{i}}}}^{2}+{\sigma_{\theta_{x_{j}}}}^{2}}$ respectively
3. Compare all models (1 to m-emitters) for a single sub-volume in which all $\theta$ and $\sigma_{\theta}$ values pass filtering steps 1-2. Use log likelihood ratio to compare models with user-defined p-value for the chi squared distribution with the degrees of freedom equal to the difference in the number model parameters [2].
4. Identify repetitive localizations and select localization with lowest relative error
   - Repetitive localizations are identified by the same procedure as that used in step 2.
   - Relative error is defined as $\left( \frac{a_{\lambda}{*\sigma}_{\theta_{\lambda}}}{\lambda_{width}} \right)^{2}+\left( \frac{a_{y}*\sigma_{\theta_{y}}}{y_{width}} \right)^{2}+\left( \frac{a_{x}{*\sigma}_{\theta_{x}}}{x_{width}} \right)^{2}$

**References**

1. Smith CS, Joseph N, Rieger B, Lidke K a (2010) Fast, single-molecule localization that achieves theoretically minimum uncertainty. Nature methods 7: 373–375. Available: http://www.pubmedcentral.nih.gov/articlerender.fcgi?artid=2862147&tool=pmcentrez&rendertype=abstract.

2. Härdle WK, Simar L (2012) Applied Multivariate Statistical Analysis. 2nd ed. Springer.
