# Supplementary material for: Multi-Color Quantum Dot Tracking Using a High-Speed Hyperspectral Line-Scanning Microscope
Source: PLoS One. 2013 May 22;8(5):e64320. doi: 10.1371/journal.pone.0064320 (PMC3661486; doi:10.1371/journal.pone.0064320)
Supplement: Text S5 — Additional details about multi-QD fitting routine. (DOCX) [file pone.0064320.s026.docx]

## Text S5. Additional Details About Multi-QD Fitting Routine.

1. Single and multi-emitter fitting performed on each sub-volume. Typically, 1-4 emitter fitting
2. Initial estimate(s)
   1. $\theta_{I}$ summation of all pixels divided by number of models (double check code)
   2. Position ($\theta_{\lambda}$*,*$\theta_{y}$*,*$\theta_{x}$)
      1. 1-emitter model: center of mass used for position estimates
      2. m-emitter model
         1. Result from select alternating iteration for model m-1 used for deflation
         2. Maximum pixel after deflation used as initial estimate for model m
   3. Spread ($\theta_{\sigma_{\lambda}}$*,* $\theta_{\sigma_{y}}$*,*$\theta_{\sigma_{x}}$)
      1. Standard values identified from localizations of 8 color of QDs on glass
      2. Typical values are 10 nm ($\theta_{\sigma_{\lambda}}$), 1.14 pixels ($\theta_{\sigma_{y}}$), and 0.80 pixels ($\theta_{\sigma_{x}}$)
   4. Zero used for $\theta_{bg}$
3. Parameter optimization
   1. Newton-Raphson iterative update method used to optimize model parameters
   2. Upper limits for single steps used to aid in convergence.
   3. Alternating iteration scheme
      1. Large parameter space for simultaneous fitting of multiple parameters leads to divergence from expected results; therefore, a scheme for limiting the number of parameters estimated simultaneously has been devised.
      2. Alternate between fixing and fitting specified model parameters. Typical, scheme depicted below:
         1. 50 iterations: optimize $\theta_{\lambda}$*,*$\theta_{y}$*,*$\theta_{x}$
         2. 50 iterations: optimize $\theta_{I}$*,*$\theta_{\lambda}$*,*$\theta_{y}$*,*$\theta_{x}$
         3. 100 iterations: optimize $\theta_{I}$*,*$\theta_{\lambda}$*,* $\theta_{\sigma_{\lambda}}$*,* $\theta_{y}$*,*$\theta_{x}$
         4. 50 iterations: optimize $\theta_{\lambda}$*,* $\theta_{\sigma_{\lambda}}$*,* $\theta_{y}$*,*$\theta_{\sigma_{y}}$*,*$\theta_{x}$*,*$\theta_{\sigma_{x}}$
