# Supplementary material for: Multi-Color Quantum Dot Tracking Using a High-Speed Hyperspectral Line-Scanning Microscope
Source: PLoS One. 2013 May 22;8(5):e64320. doi: 10.1371/journal.pone.0064320 (PMC3661486; doi:10.1371/journal.pone.0064320)
Supplement: Text S6 — Building trajectories from localizations. (DOCX) [file pone.0064320.s027.docx]

**Text S6. Building Trajectories from Localizations.**

Cost matrices are used to build trajectories in two steps: frame-to-frame and gap closing. Physical models are used to derive the costs for linking localizations, starting a new trajectory (birth), and ending a trajectory (death). The cost for connecting observations 1 and 2 is

$l_{m,n}=-log\left( P\left( x_{2},y_{2},t_{2} | x_{1},y_{1},t_{1} \right)P\left( \lambda_{2} | \lambda_{1} \right) \right)$.

Under the assumption of Brownian motion, the probability of making observation 2 ($x_{2},y_{2},t_{2}$) given observation 1 ($x_{2},y_{2},t_{2}$) is

$P\left( x_{2},y_{2},t_{2} | x_{1},y_{1},t_{1} \right)=e^{\frac{-\left( \theta_{x_{1}}-\theta_{x_{2}} \right)^{2}-\left( \theta_{y_{1}}-\theta_{y_{2}} \right)^{2}}{4D\Delta t+{2\sigma}_{1}^{2}+{2\sigma}_{2}^{2}}}$,

where

$$\sigma_{1}^{2}={\sigma_{\theta_{x_{1}}}}^{2}+{\sigma_{\theta_{y_{1}}}}^{2}$$

is the spatial localization error for observation 1 and $\sigma_{2}^{2}$ is similarly defined for observation 2. Similarly, due to the spectral emission peak, the probability of making observation 2 ($\lambda_{2})$ given observation 1 ($\lambda_{1})$ is

$P\left( \lambda_{2} | \lambda_{1} \right)=erf\left( \frac{\left| \theta_{\lambda_{1}}-\theta_{\lambda_{2}} \right|}{\sqrt{2\left( \sigma_{\theta_{\lambda_{1}}}^{2}+\sigma_{\theta_{\lambda_{2}}}^{2}+\sigma_{\lambda_{\text{jump}}}^{2} \right)}} \right)$,

where $\sigma_{\lambda_{\text{jump}}}^{2}$ accounts for the variance in the observed spectral emission peak due to spectral jumping or bluing of individual QDs. The cost for a previously unobserved QD blinking on (birth) or a currently tracked QD blinking off (death) is determined by particle density and estimated blinking rates. Building a cost matrix from physical models is discussed in more detail in a manuscript in preparation by Relich P, Cutler PJ, Huang F, Lidke KA.

The cost matrix built from costs of linking, birth, and death is treated as a linear assignment problem to link localizations into trajectories. The spectral information of localized QDs greatly improves the accuracy of trajectories and permits SPT at higher labeling densities (**Figure S14**).
