# Supplementary material for: Multi-Color Quantum Dot Tracking Using a High-Speed Hyperspectral Line-Scanning Microscope
Source: PLoS One. 2013 May 22;8(5):e64320. doi: 10.1371/journal.pone.0064320 (PMC3661486; doi:10.1371/journal.pone.0064320)
Supplement: Text S7 — Diffusion coefficient uncertainty. (DOCX) [file pone.0064320.s028.docx]

**Text S6. Diffusion Coefficient Uncertainty.**

Assuming Brownian motion, the mean squared displacement for a 2D trajectory is defined as

$\left\langle r^{2} \right\rangle=4D\Delta t$,

where $D$ is the diffusion coefficient, and $\Delta t$ is the time lag between observations. The probability distribution of displacements given some underlying diffusion is

$P\left( r | D \right)=\frac{r}{2D\Delta t}e^{\left( \frac{-r^{2}}{4D\Delta t} \right)}$.

Given $N$ observed displacements ($\vec{r})$ and an estimator of $D$ that maximizes the likelihood function

$L\left( \theta_{D} | \vec{r} \right)=\prod_{k=1}^{N} \frac{r_{k}}{2D\Delta t}e^{\left( \frac{-r_{k}^{2}}{2D\Delta t} \right)}$,

information theory uses the Fisher Information Matrix

$$I\left( \theta_{D} \right)=-E\left[ \frac{\partial^{2}log\left( L\left( \theta_{D} | \vec{r} \right) \right)}{\partial^{2}\theta_{D}} \right]$$

to define the minimum variance (CRB) with which $\theta_{D}$ can be estimated

$\sigma_{\theta_{D}}^{2}={I\left( \theta_{D} \right)}^{-1}=\frac{D^{2}}{N}$.

The average number of observed particles in a region of interest $A$ at a particle density $\rho$ over $n$ frames is

$\bar{\text{x}}=A\rho n$.

$\bar{\text{x}}$ is directly related to the number of observed displacements ($N$); therefore,

$\sigma_{\theta_{D}}\approx\frac{D}{\sqrt{A\rho n}}$.

The difference between $\bar{\text{x}}$ and $N$ is dependent on the number of trajectories.
