# Supplementary material for: Multi-Color Quantum Dot Tracking Using a High-Speed Hyperspectral Line-Scanning Microscope
Source: PLoS One. 2013 May 22;8(5):e64320. doi: 10.1371/journal.pone.0064320 (PMC3661486; doi:10.1371/journal.pone.0064320)
Supplement: Text S8 — Theoretical performance of line and point scanning systems. (DOCX) [file pone.0064320.s029.docx]

**Text S8. Theoretical Performance of Line and Point Scanning Systems.**

The number of photons that can be captured in a given frame limits the localization accuracy for each emitter in each frame for a given optical system. Fluorescent dyes and quantum dots have a finite excited state mean lifetime, τ, which ultimately bounds the emission rate to 1/τ in the limit of very large excitation intensities. This maximum emission rate is commonly referred to as the saturation rate (i.e. a sample of emitters is exposed to excitation light of such high intensity that essentially none are ever in the ground state as they are immediately excited).

For emission wavelength *λ_em_*, numerical aperture *NA*, overall optical efficiency of the emission path *γ*, emission rate *A*, and exposure time *t*, the theoretical best possible localization accuracy [1] is given by:

$$\delta= \frac{\lambda_{em}}{2\pi(NA)\sqrt{\gamma At}}$$

In the limit of excited state saturation, *A* = 1/*τ* photons/sec,

$$\delta= \frac{\lambda_{em}}{2\pi(NA)\sqrt{\frac{\gamma t}{\tau}}}$$

If we desire some approximate localization accuracy *δ*, and wish to find the minimum exposure time required to achieve that, we can rearrange the equation, solving for *t*:

$$t= \left( \frac{\lambda_{em}}{2\pi(NA)\delta} \right)^{2}\frac{\tau}{\gamma}$$

If we consider a single emitter species, say quantum dot 625, with a peak emission wavelength of 625 nm and mean excited state lifetime of 20 ns, a system with a numerical aperture of 1.2, and overall optical transmission efficiency of 0.04 (including detector efficiency and solid angle acceptance of the objective), then a desired localization accuracy of 10 nm would require an exposure time of ~31 μs for that emitter.

A point scanning system would use the required exposure time as the pixel dwell time (the amount of time the excitation beam must stay at each (*x*,*y*) pixel position on the sample). Since a point scanning system raster scans the point over the sample region of interest one (*x*,*y*) pixel position at a time, the maximum overall frame rate is

$$r_{point}=\frac{1}{\left( t*N_{x}*N_{y} \right)}$$

where *N_x_* is the number of pixels in *x* and *N_y_* is the number of pixels in *y*. A line scanning system will have the same dwell time, but this will be for an entire row (or column) of pixels, so the frame rate is

$$r_{line}=\frac{1}{\left( t*N_{x} \right)}$$

In the case where we have a region of interest that is composed of 128×128 spatial pixels, for the desired localization accuracy and system described above, *r_point_* is ~2 fps and *r_line_* is ~250 fps. This illustrates not only a performance advantage of line scanning over point scanning systems, but also that the theoretical frame rate is much higher than currently possible with camera technology that is available at the time of this writing, and this is a key area of potential improvement as camera speeds increase.

**References**

1. Ober RJ, Ram S, Ward ES (2004) Localization accuracy in single-molecule microscopy. Biophysical journal 86: 1185–1200. Available: http://www.pubmedcentral.nih.gov/articlerender.fcgi?artid=1303911&tool=pmcentrez&rendertype=abstract.
