# Supplementary material for: Multi-Color Quantum Dot Tracking Using a High-Speed Hyperspectral Line-Scanning Microscope
Source: PLoS One. 2013 May 22;8(5):e64320. doi: 10.1371/journal.pone.0064320 (PMC3661486; doi:10.1371/journal.pone.0064320)
Supplement: Table S1 — Description of Gaussian fitting variables. (DOCX) [file pone.0064320.s030.docx]

Table S1. Description of Gaussian Fitting Variables.

| Symbol | Description |
| --- | --- |
| *General Variables* | |
| $\lambda$ | Spectral position |
| $y$ | Spatial position along the line |
| $x$ | Spatial position in the scan direction |
| $\lambda_{width}$ | Width of a spectral pixel |
| $\mu_{k}$ | Counts in pixel *k* of single QD model |
| $\mu_{k_{All}}$ | Counts in pixel *k* of *N* QD model with background |
| $d_{k}$ | Counts in pixel *k* of experimental image |
| $\sigma_{\theta}$ | Theoretical error (standard deviation) for model parameter as approximated by the Cramér Rao Bound |
|  |  |
| *Model Parameters for an individual QD* | |
| $\theta_{I}$ | Photon counts |
| $\theta_{\lambda}$ | Position of spectral emission peak of QD |
| $\theta_{\sigma_{\lambda}}$ | Spectral spread (standard deviation) of QD |
| $\theta_{y}$ | Position of QD along y (line) dimension |
| $\theta_{\sigma_{y}}$ | Microscope point spread function in y (line) dimension |
| $\theta_{x}$ | Position of QD along x (scan) dimension |
| $\theta_{\sigma_{x}}$ | Microscope point spread function in the x (scan) dimension |
| $\theta_{bg}$ | Background offset |
