# Supplementary material for: Multi-Color Quantum Dot Tracking Using a High-Speed Hyperspectral Line-Scanning Microscope
Source: PLoS One. 2013 May 22;8(5):e64320. doi: 10.1371/journal.pone.0064320 (PMC3661486; doi:10.1371/journal.pone.0064320)
Supplement: Table S2 — Single QD characterization. (DOCX) [file pone.0064320.s031.docx]

Table S2. Single QD Characterization

Statistics for results shown in **Figure S12** are summarized in this table. Additional descriptions of the model parameters are included in **Table S1**.

| **Invitrogen QD specie** | **Number of localizations** | $\boldsymbol{\theta}_{\boldsymbol{\lambda}}$ **(nm)**  *mean* | $\boldsymbol{\theta}_{\boldsymbol{\lambda}}$ **(nm)**  *stdev* | $\boldsymbol{\theta}_{\boldsymbol{\sigma}_{\boldsymbol{\lambda}}}$ **(nm)**  *mean* | $\boldsymbol{\theta}_{\boldsymbol{\sigma}_{\boldsymbol{\lambda}}}$ **(nm)**  *stdev* | $\boldsymbol{\theta}_{\boldsymbol{I}}$ **(counts)**  *mean* |
| --- | --- | --- | --- | --- | --- | --- |
| **QD525** | 1527 | 525 | 7 | 8 | 2 | 208 |
| **QD565** | 23053 | 563 | 9 | 9 | 2 | 411 |
| **QD585** | 28777 | 587 | 10 | 9 | 2 | 541 |
| **QD605** | 36216 | 607 | 7 | 8 | 2 | 956 |
| **QD625** | 52666 | 622 | 9 | 10 | 2 | 800 |
| **QD655** | 17147 | 653 | 8 | 11 | 2 | 1218 |
| **QD705** | 25731 | 696 | 17 | 17 | 4 | 412 |
| **QD800** | 5795 | 733 | 15 | 15 | 4 | 209 |
